# Multi-omics data integration using ratio-based quantitative profiling with Quartet reference materials

**DOI:** 10.1038/s41587-023-01934-1

**Published:** 2023-09-07

**Authors:** Yuanting Zheng, Yaqing Liu, Jingcheng Yang, Lianhua Dong, Rui Zhang, Sha Tian, Ying Yu, Luyao Ren, Wanwan Hou, Feng Zhu, Yuanbang Mai, Jinxiong Han, Lijun Zhang, Hui Jiang, Ling Lin, Jingwei Lou, Ruiqiang Li, Jingchao Lin, Huafen Liu, Ziqing Kong, Depeng Wang, Fangping Dai, Ding Bao, Zehui Cao, Qiaochu Chen, Qingwang Chen, Xingdong Chen, Yuechen Gao, He Jiang, Bin Li, Bingying Li, Jingjing Li, Ruimei Liu, Tao Qing, Erfei Shang, Jun Shang, Shanyue Sun, Haiyan Wang, Xiaolin Wang, Naixin Zhang, Peipei Zhang, Ruolan Zhang, Sibo Zhu, Andreas Scherer, Jiucun Wang, Jing Wang, Yinbo Huo, Gang Liu, Chengming Cao, Li Shao, Joshua Xu, Huixiao Hong, Wenming Xiao, Xiaozhen Liang, Daru Lu, Li Jin, Weida Tong, Chen Ding, Jinming Li, Xiang Fang, Leming Shi

**Affiliations:** 1 State Key Laboratory of Genetic Engineering, School of Life Sciences, Human Phenome Institute and Shanghai Cancer Center, Fudan University, Shanghai, China; 2 Greater Bay Area Institute of Precision Medicine, Guangzhou, China; 3 National Institute of Metrology, Beijing, China; 4 National Center for Clinical Laboratories, Institute of Geriatric Medicine, Chinese Academy of Medical Sciences, Beijing Hospital, Beijing, China; 5 Vazyme Biotech Co. Ltd., Nanjing, China; 6 MGI, BGI-Shenzhen, Shenzhen, China; 7 Zhangjiang Center for Translational Medicine, Shanghai Biotecan Medical Diagnostics Co. Ltd., Shanghai, China; 8 Novogene Bioinformatics Institute, Beijing, China; 9 Metabo-Profile Biotechnology (Shanghai) Co. Ltd., Shanghai, China; 10 Calibra Diagnostics, Hangzhou, China; 11 Nextomics Biosciences Institute, Wuhan, China; 12 Genome Decoding Institute, Nantong, China; 13 Institute for Molecular Medicine Finland (FIMM), University of Helsinki, Helsinki, Finland; 14 EATRIS ERIC–European Infrastructure for Translational Medicine, Amsterdam, the Netherlands; 15 Key Laboratory of Bioanalysis and Metrology for State Market Regulation, Shanghai Institute of Measurement and Testing Technology, Shanghai, China; 16 Division of Bioinformatics and Biostatistics, National Center for Toxicological Research, US Food and Drug Administration, Jefferson, AR USA; 17 Office of Oncologic Diseases, Office of New Drugs, Center for Drug Evaluation and Research, US Food and Drug Administration, Silver Spring, MD USA; 18 Shanghai Institute of Immunity and Infection, Chinese Academy of Sciences, Shanghai, China; 19 International Human Phenome Institutes (Shanghai), Shanghai, China

**Keywords:** Standardization, Quality control, Data integration

## Abstract

Characterization and integration of the genome, epigenome, transcriptome, proteome and metabolome of different datasets is difficult owing to a lack of ground truth. Here we develop and characterize suites of publicly available multi-omics reference materials of matched DNA, RNA, protein and metabolites derived from immortalized cell lines from a family quartet of parents and monozygotic twin daughters. These references provide built-in truth defined by relationships among the family members and the information flow from DNA to RNA to protein. We demonstrate how using a ratio-based profiling approach that scales the absolute feature values of a study sample relative to those of a concurrently measured common reference sample produces reproducible and comparable data suitable for integration across batches, labs, platforms and omics types. Our study identifies reference-free ‘absolute’ feature quantification as the root cause of irreproducibility in multi-omics measurement and data integration and establishes the advantages of ratio-based multi-omics profiling with common reference materials.

## Abstract (web summary)

Multi-omics data are readily integrated using quartet reference materials.

## Main

Multi-omics profiling is a new approach in which molecular phenomics data across multiple omics layers, including genomes, epigenomes, transcriptomes, proteomes and metabolomes, of a sample or set of samples are fully measured, analyzed and integrated from the same set of samples on a genome scale^1–3^. Multi-omics profiling quantifies biologically different signals across complementary omics layers and can therefore explore the intricacies of interconnections between multiple layers of biological molecules and identify system-level biomarkers^4–8^. Technology innovations and cost reduction have empowered increasingly large-scale multi-omics studies for data collection on the same group of individuals, providing a unique opportunity to fully understand and yield high-level insights into human diseases in a holistic fashion^9–14^.

Multi-omics data integration can be classified into sample and feature integration^15^. When the objective is to find relationships among samples, the common multi-omics integration strategy is to use a data-driven clustering approach or classify biological samples by combining complementary information, followed by extracting system-level biologically differentiated networks for endpoints such as wellness or disease subtyping^16–19^ or longitudinal trajectories^20–22^. When the objective is to look at measured features, multilayered molecular networks are identified so as to reveal the perturbed signatures and potential actionable targets for disease prevention and treatment^23–31^. Assigning accurate sample groups and extracting true biological networks is challenging owing to the complexity and diversity of multi-omics datasets^15,32,33^. Moreover, large-scale consortia-based multi-omics data are often generated across platforms, labs and batches, creating unwanted variations and multiplying the complexities. Therefore, efficient data integration is essential for reliable multi-omics studies^32^.

Data integration in large-scale multi-omics studies usually falls into two categories of application scenarios: horizontal and vertical^34^. Horizontal (within-omics) integration, that is, integration of diverse datasets from a single omics type, aims to combine multiple datasets from the same omics type across multiple batches, technologies and labs for downstream analysis. Unwanted variations can result in systematic deviations known as batch effects, confounded with critical study factors^35,36^. Currently, various horizontal integration methods are available for bulk and single-cell omics data^37–39^. Selection of horizontal integration methods based on arbitrary visualizations of integrated datasets is challenging owing to the lack of ground truth and objective quality control (QC) metrics for method selection. Vertical (cross-omics) integration, that is, integration of diverse datasets from multiple omics types, aims to combine multiple omics datasets with different modalities from the same set of samples, followed by design of appropriate downstream analysis to identify accurate sample groups or multilayered and interconnected networks of biomolecular features^6,34,40,41^.

Devising proper vertical integration strategies for sample clustering or feature identification is challenging in multi-omics profiling. First, different technologies result in varying numbers of features and statistical properties, which can have a strong influence on the integration step to appropriately select and weigh different modalities. Second, each omics dataset has intrinsic technological limitations and noise structures. Combining multi-omics datasets also multiplies all the technical noise across different technologies, making it more difficult to integrate multiple datasets. Third, many multi-omics data integration algorithms and software programs have been developed on the basis of different statistical principles and assumptions^42–44^. Each multi-omics integration method can report a solution, but assessing its reliability is difficult owing to the lack of multi-omics ‘ground truth’ and QC methods for these complex processes.

Multi-omics reference materials and relevant QC metrics are required for quality assessment of each type of omics measurement and its horizontal integration before successful multi-omics-level vertical data integration^45–48^. Unrelated reference materials have been widely used as ground truth when evaluating technologies for the same omics type, such as genomic DNA^49,50^, tumor–normal paired DNA^51–53^, RNA, protein or metabolite reference materials^54–57^. However, multi-omics profiling requires measurement of multiple types of omics data from the same set of interconnected reference samples, thus allowing for assessment of the ability to distinguish different reference samples with integrated datasets. Moreover, DNA, RNA, protein and metabolite reference materials should be prepared simultaneously, which can provide ‘built-in truth’ (the central dogma of information flow from DNA to RNA to protein) for validating the hierarchical relationship among identified features. Therefore, publicly accessible and well-characterized multi-omics reference materials at the genome scale are urgently needed^47^.

Notably, QC metrics relevant to research purposes are also critically important for assessing the quality of multi-omics profiling. Precision and recall are widely used QC metrics for qualitative genomic variant calling^58–60^, whereas correlation coefficients are widely used for quantitative omics profling^55,56,61–64^. However, multi-omics profiling is an integrated process; therefore, the QC process should be performed on the basis of the entire sample-to-result pipeline. Integrating multi-omics information for more robust sample classifiers and multilayered interconnected molecular signatures is the major goal for multi-omics profiling. Therefore, QC metrics should be related to these two critical research objectives and should be suitable for evaluating the performance of each omics type ranging from data generation to multi-omics data integration.

We launched the Quartet Project (https://chinese-quartet.org/) to provide multi-omics ground truth as well as best practices for the QC and data integration of multi-omics profiling. The Quartet multi-omics reference material suites include references of DNA, RNA, protein and metabolites developed from B-lymphoblastoid cell lines (LCLs) derived from a quartet family of parents and monozygotic twin daughters and were designed to objectively evaluate the wet-lab proficiency in data generation and reliability of computational methods for horizontal integration of data of the same omics type and for vertical integration of data of multiple omics types. A broad collection of the Quartet multi-omics data generated from key technologies provides rich resources for evaluating the performance of new labs, platforms, protocols and analytical tools. On the basis of the pedigree information for the Quartet samples, performance of horizontal and vertical data integration can be objectively evaluated, which provides unique insights into the commonly used multi-omics integration strategies. We also developed a user-friendly data portal for the community to conveniently use and improve the Quartet resources (https://chinese-quartet.org/). Most importantly, our study identifies absolute feature quantification as the root cause of irreproducibility in multi-omics measurement and data integration and urges a paradigm shift from absolute to ratio-based quantitative multi-omics profiling.

## Results

### Overview of the Quartet Project

**Fig. 1:**
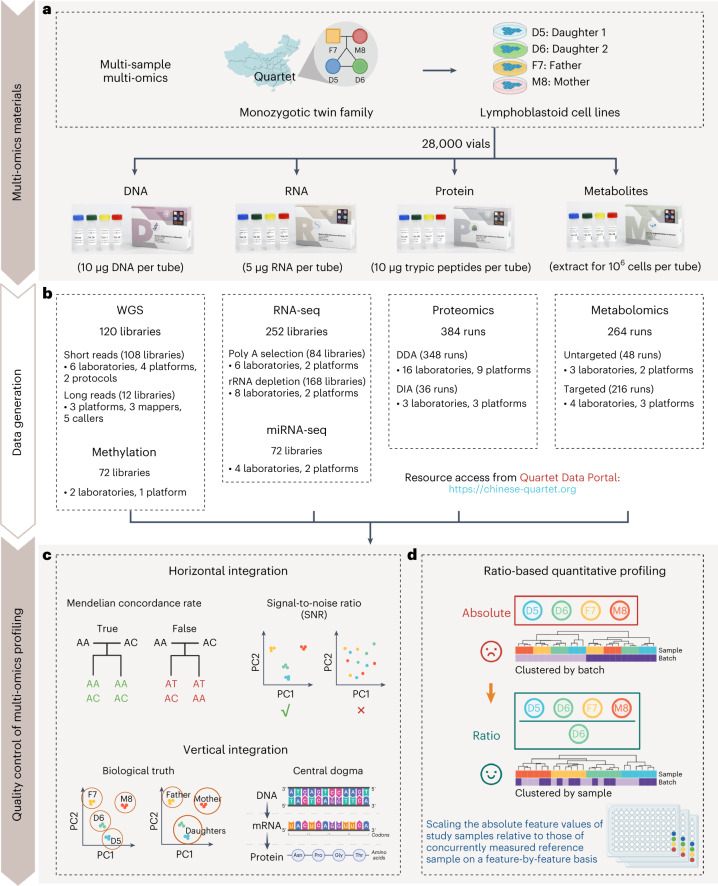
Overview of the Quartet Project. **a**, Design and production of Quartet family-based multi-omics reference material suites. **b**, Data generation across multiple platforms, labs, batches and omics types. DDA, data-dependent acquisition; DIA, data-independent acquisition; WGS, whole-genome sequencing. **c**, QC metrics for horizontal (within-omics) integration include the Mendelian concordance rate and SNR, which are also applicable to wet-lab proficiency testing. Two types of QC metrics for vertical (cross-omics) integration were developed that assess the ability to detect cross-omics feature relationships that follow the central dogma and the ability to classify samples into either four phenotypically different groups (D5–D6–F7–M8) or three genetically driven clusters (daughters–father–mother). **d**, Ratio-based scaling using common reference materials empowers horizontal and vertical integration.

**Extended Data Fig. 1:**
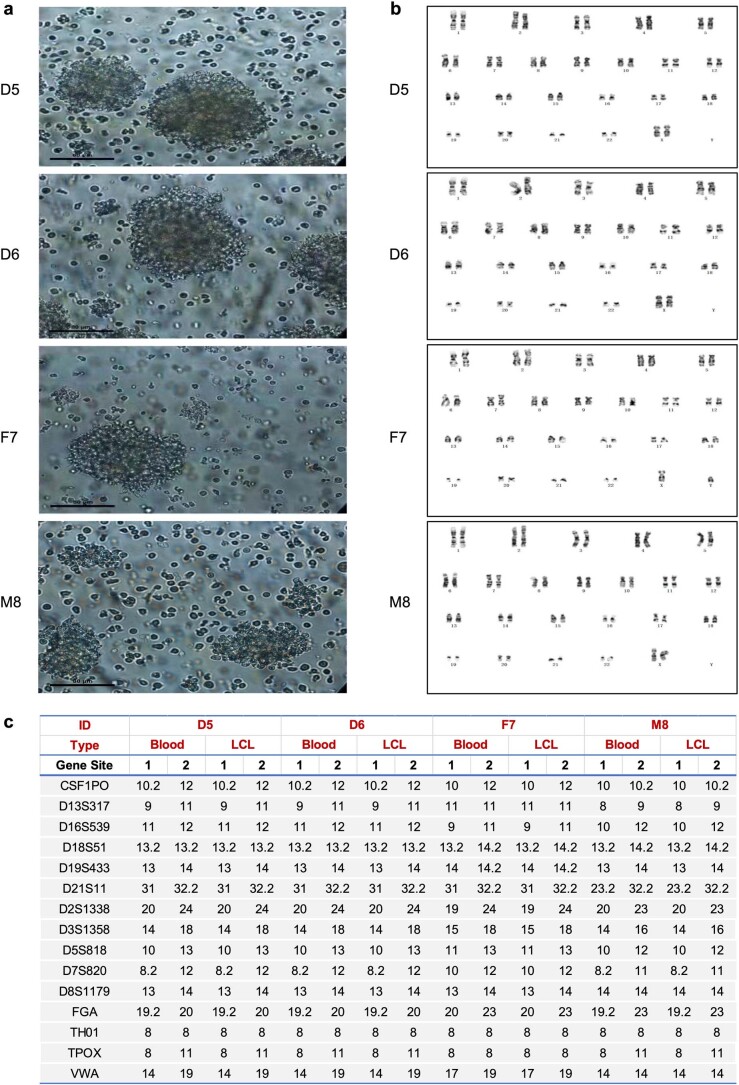
Characterization of the Quartet B-lymphoblastoid cell lines (LCLs). **a**, Quartet LCLs were cultured in suspension with typical cell clusters. At least six images were captured under phase-contrast microscopy (X20), and representative images were shown (scaled bar 80 µm). **b**, Normal karyotypes of the LCLs were shown. **c**, 15 STR loci were used for identification of Quartet monozygotic twins’ family. Importantly, there were no differences between results from DNAs isolated from LCLs and primary blood.

The Quartet Project provides the community with multi-omics reference materials and reference datasets for QC and data integration in increasingly large-scale multi-omics studies (Fig. 1a). Suites of large quantities of multi-omics reference materials (DNA, RNA, protein and metabolites) were simultaneously established from the same immortalized LCLs of a Chinese Quartet family from the Fudan Taizhou Cohort^65^ (Extended Data Fig. 1), including the father (F7), mother (M8) and monozygotic twin daughters (D5 and D6). As summarized in Extended Data Table 1, each reference material was stocked in more than 1,000 vials. These reference materials are suitable for a wide range of multi-omics technologies, including DNA sequencing, DNA methylation analysis, RNA sequencing (RNA-seq), microRNA sequencing (miRNA-seq), and liquid chromatography and tandem mass spectrometry (LC–MS/MS)-based proteomics and metabolomics. Notably, the DNA and RNA reference material suites have been approved by China’s State Administration for Market Regulation as the First Class of National Reference Materials (GBW 099000–GBW 099007) and are being used extensively for proficiency testing and method validation.

For comprehensive performance evaluation, the Quartet multi-omics reference material suites were profiled across commonly used multi-omics platforms, including seven DNA sequencing platforms, one DNA methylation platform, two RNA-seq platforms, two miRNA-seq platforms, nine LC–MS/MS-based proteomics platforms and five LC–MS/MS-based metabolomics platforms (Fig. 1b). For performance evaluation, three technical replicates for each reference material were measured in each lab, except for the long-read DNA sequencing platforms, for which only one replicate was sequenced for each platform. Supplementary Table 1 summarizes the Quartet multi-omics datasets for the real-world assessment of commonly used multi-omics technologies. All the data can be accessed from the Quartet Data Portal (https://chinese-quartet.org/), which provides a landscape of data quality for each type of omics profiling.

The Quartet Project provides a set of metrics for QC and data integration in multi-omics profiling. For generation and horizontal integration of data from each omics type, the Quartet built-in QC metrics, that is, the Mendelian concordance rate for genomic variant calls and signal-to-noise ratio (SNR) for quantitative omics profiling, enable proficiency testing on a whole-genome scale using the Quartet reference materials. In addition, the Quartet multi-omics design provides two types of QC metrics to evaluate the reliability of vertical integration. One assesses the ability to correctly classify the Quartet samples into both four different individuals (daughter1–daughter2–father–mother) and three genetically driven clusters (daughters–father–mother), which is related to the multi-omics research purpose of sample clustering. Another QC metric assesses the ability to correctly identify cross-omics feature relationships that follow the central dogma (information flow from DNA to RNA to protein) and can be used to evaluate the reliability of correlation-based multi-omics integration (Fig. 1c). We propose ratio-based profiling using common reference materials to empower horizontal and vertical omics data integration. Ratio-based data were derived by scaling the absolute feature values of study samples (such as D5, F7 and M8) relative to those of a concurrently measured reference sample (such as D6) on a feature-by-feature basis (Fig. 1d).

**Extended Data Fig. 2:**
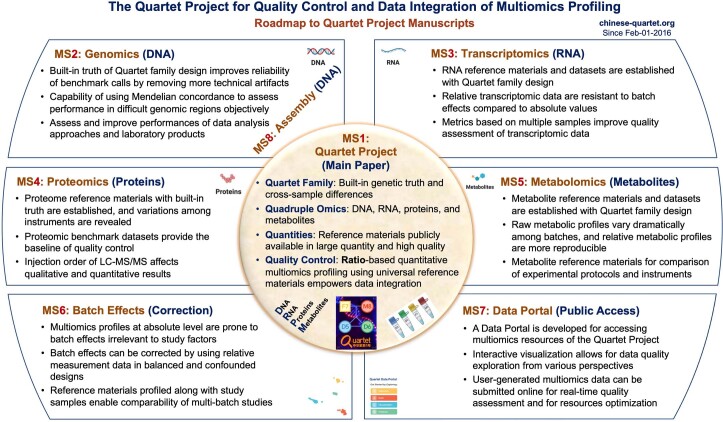
Roadmap to the Quartet Project manuscripts. **MS1**: Quartet project overview and main findings; **MS2/3/4/5**: Genomics / Transcriptomics / Proteomics / Metabolomics reference materials and reference datasets; **MS6**: Batch effects and correction; **MS7**: Data portal for public access of Quartet Project resources; **MS8**: Haplotype-resolved assemblies.

In this article, we provide an overview of the Quartet Project and propose a ratio-based quantitative profiling approach for multi-omics data integration using Quartet reference datasets across multiple omics types, platforms, batches and labs (Extended Data Fig. 2). Four accompanying papers detail the establishment of the DNA^66^, RNA^67^, protein^68^ and metabolite^69^ reference materials, reference datasets and QC methods for each type of omics profiling. Haplotype-resolved assemblies and a variant benchmark have also been provided^70^. Another paper^71^ is dedicated to benchmarking batch effect correction algorithms (BECAs) using the Quartet multi-omics data. We have also developed the Quartet Data Portal (https://chinese-quartet.org/)^72^ for the community to conveniently access and share the Quartet multi-omics resources according to the regulations of the Human Genetic Resources Administration of China.

### Wet-lab proficiency in omics data generation varies

Before data integration, the proficiency in data generation for each type of omics data was assessed. Except for the long-read sequencing platforms, the reference materials were profiled within a batch in a lab in three replicates for each of the four samples (donors). For long-read sequencing, one replicate for each reference material was sequenced and the resulting data were analyzed using 11 pipelines; therefore, the performance evaluation was conducted only at the level of analytical procedures. Details on data generation and analysis are provided in the Methods.

**Fig. 2:**
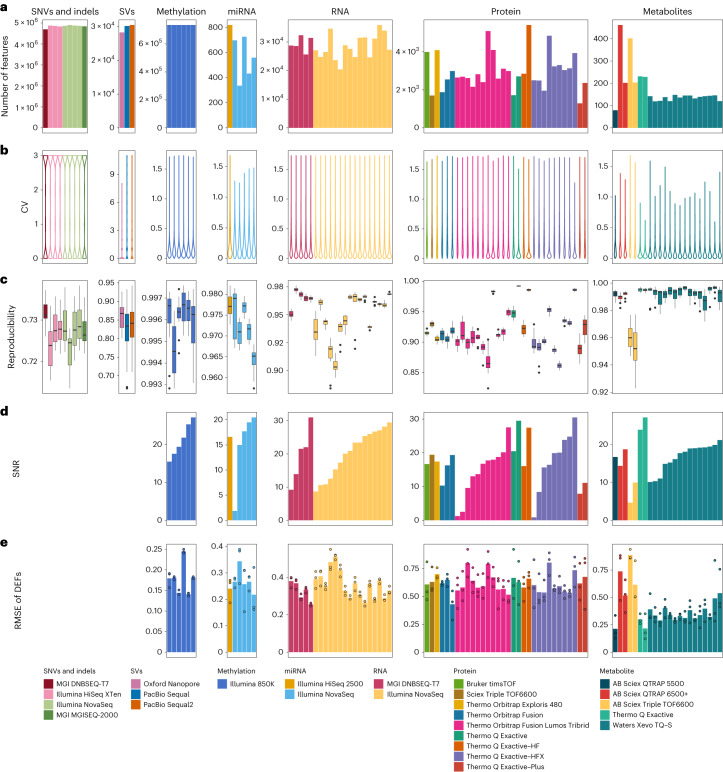
Wet-lab proficiency in omics data generation varies. **a**, The number of features detected from each dataset generated in different labs using different platforms. **b**, Distribution of the number of experiments supporting genomic variant calling or CV in quantitative omics profiling from technical replicates (analytical repeats in SV calling and library repeats for the others) within a batch. **c**, Technical reproducibility from three replicates within a batch, calculated as the Jaccard index for small variant calling and Pearson correlation coefficient (*r*) for quantitative omics profiling (*n* = 12). For SV call sets, technical reproducibility was defined as the Jaccard index between different analytical repeats (Oxford Nanopore, *n* = 28; PacBio Sequal, *n* = 55; PacBio Sequal2, *n* = 55). The box plots display the distribution of data, with the median represented by the line inside the box and the interquartile range represented by the box. Whiskers extend to 1.5× the interquartile range. **d**, SNR based on the Quartet multi-sample design (4 samples × 3 replicates per batch). **e**, RMSE of high-confidence DEFs. Dots represent RMSE values for the D5–F7, D5–M8 and F7–M8 pairs in each batch (*n* = 3), while the bar plots present the corresponding mean values.

QC metrics for evaluation of objective performance are critically important. The number of measured features, coefficient of variation (CV) and technical reproducibility are widely used QC metrics across different omics platforms and were used in our study for cross-omics performance comparisons. As shown in Fig. 2, the number of features measured for each omics type varied by several orders of magnitude, from 60 metabolites to 4.8 million small DNA variants (single-nucleotide variants (SNVs) and indels) per sample (Fig. 2a). Within each omics type, the number of features detected varied among batches and labs. The reproducibility of detected features in each omics profiling type was evaluated using the number of replicates supporting a variant call for genomics and the CV among technical replicates within a batch in quantitative omics profiling (Fig. 2b). Most SNVs were supported by all three library replicates within the batch, whereas the number of analytical repeats supporting a structural variant (SV) call greatly varied. For quantitative omics profiling, the CVs of most quantified features were below 30%. In addition, the reproducibility of technical replicates was also evaluated at the individual sample level. Reproducibility was calculated as the Jaccard index from three library repeats within a batch. For the short-read sequencing platforms, all Jaccard index values were above 93%. Moreover, the reproducibility of SVs from 11 call sets using different analytical pipelines was between 80% and 90%. Nanopore was found to be more reproducible than PacBio among the long-read sequencing platforms. The reproducibility of quantitative omics profiling was calculated as the Pearson correlation coefficient (Pearson’s *r*) of technical replicates within a batch. The *r* values from all labs and metabolomic platforms were above 95%, indicating high reproducibility in metabolomic profiling for the same sample. However, the *r* values for repeated measurements of the same sample were between 88.42% and 97.62% for transcriptomics and between 82.37% and 99.34% for proteomics (Fig. 2c).

On the basis of the Quartet multi-sample design, we defined two QC metrics to measure the ability to identify intrinsic biological differences among various groups of samples, a key objective of omics profiling. The Quartet-based SNR metric is the ratio of inter-sample differences (that is, ‘signal’) to intra-sample differences among technical replicates *(*that is, ‘noise’). It is calculated as the ratio of the average distance between the Quartet samples to the average distance between technical replicates of the same sample (see Methods for details). For a measurement method with high resolution in differentiating biologically different groups of samples, the inter-sample differences of the Quartet samples should be much larger than the variation among technical replicates for the same sample. Principal-component analysis (PCA) showed clear separation among the Quartet samples (D5, D6, F7 and M8) for high-quality profiling experiments (Supplementary Fig. 1a) but not for low-quality profiling experiments (Supplementary Fig. 1b). Strikingly, high variabilities in intra-batch data quality were observed in each omics platform (Fig. 2d), especially for the quantitative omics platforms, including for methylomics (SNR range of 15.5–27.1, s.d. = 4.5), transcriptomics (SNR range of 8.7–31.0, s.d. = 7.1), miRNA profiling (SNR range of 1.9–20.5, s.d. = 6.8), proteomics (SNR range of 0.9–30.5, s.d. = 7.5) and metabolomics (SNR range of 4.6–27.1, s.d. = 5.1). Moreover, high variabilities of proficiency in data generation were evident for each technology platform. For example, both high and low SNRs were observed in RNA-seq for the Illumina and BGI platforms, but the average SNRs across multiple batches were very close for the two sequencing platforms (20.39 versus 19.54, *P* = 0.84). Similarly, high variabilities in SNR were observed within each MS platform for proteomics or metabolomics profiling. These results implied that the inherent proficiency of an individual wet lab, instead of a specific platform itself, was a more important factor affecting the reliability of data generation for each omics type.

**Extended Data Fig. 3:**
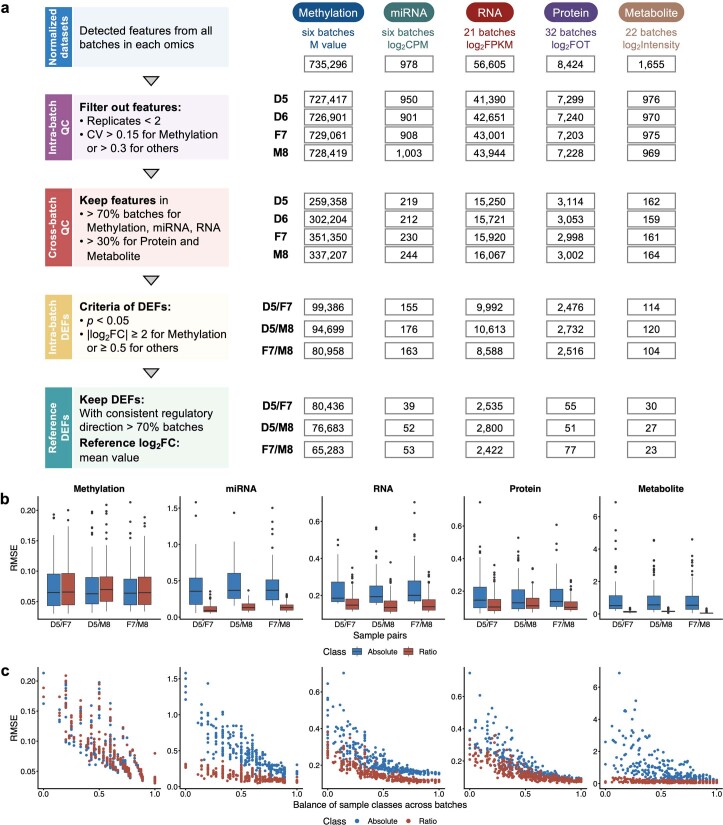
Ratio-based scaling promotes accurate identification of differentially expressed features. **a**, Workflow for the construction of reference datasets of differentially expressed features. Reference datasets were constructed according to the following steps: (1) Identifying detectable multi-omics features and per-sample normalization. (2) Intra-batch quality control. Features that were not detectable or had low technical reproducibility were filtered out. (3) Cross-batch quality control. Features detectable in a sufficient number of batches were retained. (4) Calculating intra-batch differentially expressed features (DEFs) using t-test analysis. DEFs were classified as up- or down-regulated based on the positive or negative sign of the log2 fold change. (5) Voting based on the regulatory directionality (up or down) to screen the high confidence DEFs. **b**, Box plots of RMSE of the DEFs of horizontal integration data at absolute level (Blue) and ratio level (Red) based on the reference datasets. Data were sampled 100 times per pair of samples. The box plots display the distribution of data with the median represented by the line inside the box and the interquartile range (IQR) represented by the box. Whiskers extend to 1.5× the interquartile range. **c**, Scatter plots between RMSE when integrating at absolute (Blue) and ratio (Red) levels and the degrees of sample class-batch balance.

In addition, we constructed high-confidence reference datasets (Supplementary Table 2) of differentially expressed features (DEFs) in terms of the level of differential expression between pairs of samples (D5–F7, D5–M8 and F7–M8 pairs) for each quantitative omics profiling type using a consensus-based integration strategy (Extended Data Fig. 3a). Root mean square error (RMSE) was used to quantitatively evaluate the consistency of a test dataset with the high-confidence reference dataset (Fig. 2e).

We further explored the relationships between SNR and the number of detected features, the reproducibility of features, the reproducibility of technical replicates and the RMSE of DEFs to evaluate data quality in quantitative omics profiling (Supplementary Fig. 2). These data suggested that none of the widely used QC metrics (number of measured features, CV of measured features and correlation of technical replicates) based on a single reference sample guarantee high resolution (SNR) in identifying inherent differences (that is, biological signals) among various biological sample groups. Therefore, multi-sample-based QC metrics are needed to identify labs with low proficiency in detecting intrinsic biological differences among sample groups.

### Ratio-based scaling enables horizontal integration

In large-scale omics studies, the reliability of horizontal integration of omics datasets across different platforms, labs or batches for the same omics type is essential. We propose a ratio-based scaling approach (for example, D5, F7 and M8 as study samples) using common reference material(s) (for example, D6) to enable horizontal integration of diverse datasets from the same omics type.

**Fig. 3:**
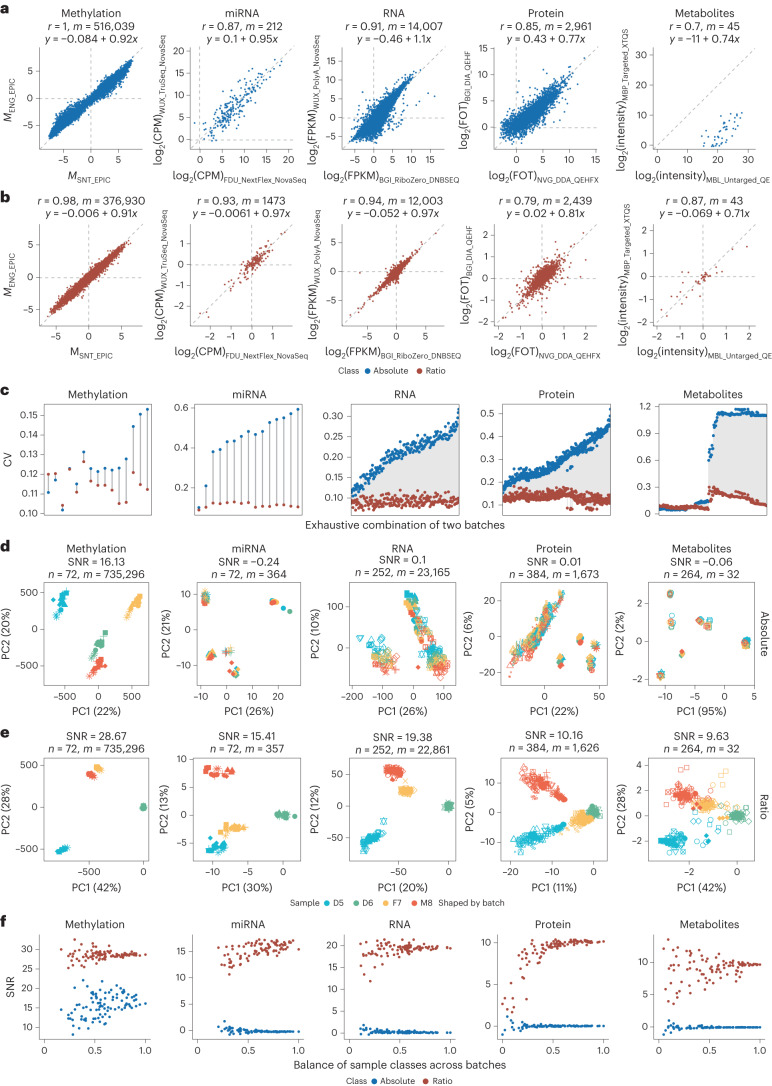
Ratio-based scaling enables horizontal integration. **a**,**b**, Scatterplots of the feature abundance of inter-batch D5 samples in methylation, miRNA-seq, RNA-seq, proteomics and metabolomics datasets at the absolute level (raw data; **a**) and ratio level (ratio scaling to the D6 sample; **b**). The *x* and *y* axes show the average expression of the three D5 technical replicates from the two best quality batches from different labs (ranked by SNR). At the absolute level, features with a CV less than 0.2 for the technical replicates of D5 in both batches were retained; at the ratio level, features with a CV less than 0.2 for the technical replicates of D5 and D6 in both batches were retained. *r* denotes the Pearson correlation coefficient, and *m* denotes the number of features. Linear fits were performed on the basis of the feature abundance. **c**, Lollipop plots of CV in feature abundance for six D5 samples across two batches. The *x* axis represents the exhaustive two-by-two combination of all batches for each omics type. **d**,**e**, PCA plots of horizontal integration of all batches of methylation, miRNA-seq, RNA-seq, proteomics and metabolomics datasets at the absolute level (**d**) and ratio level (**e**). *n* denotes the number of samples, and *m* denotes the number of features. **f**, Scatterplots between SNR and degree of sample class-batch balance. Blue, absolute level; red, ratio level.

Technical variations are dominant during horizontal integration of the Quartet data at the absolute level. For methylation array data represented as *M* value, miRNA-seq data represented as log_2_(counts per million mapped reads (CPM)), RNA-seq data represented as log_2_(fragments per kilobase of transcript per million mapped reads (FPKM)), proteomics data represented as log_2_(fraction of total (FOT)) and metabolomics data represented as log_2_(intensity), systematic deviations were observed between two technical replicates (D5) from different batches after horizontal integration (Fig. 3a). The intercepts for the fitted lines for each scatterplot ranged from –0.084 to –11, with integrability being the worst for absolute metabolomics profiling. However, after scaling the absolute feature values of D5 relative to those for a concurrently measured D6 sample on a feature-by-feature basis, the systematic deviations for each omics profiling type were reduced. The intercepts for all the fitted lines decreased; in particular, the intercept decreased markedly from –11 (absolute) to –0.069 (ratio) for metabolomics profiling (Fig. 3b). In addition, the CV values of six technical replicates of D5 samples from an exhaustive combination of two batches of datasets were mostly decreased at the ratio level except for some combinations of metabolomics profiling from the same lab (Fig. 3c and Supplementary Fig. 3).

**Extended Data Fig. 4:**
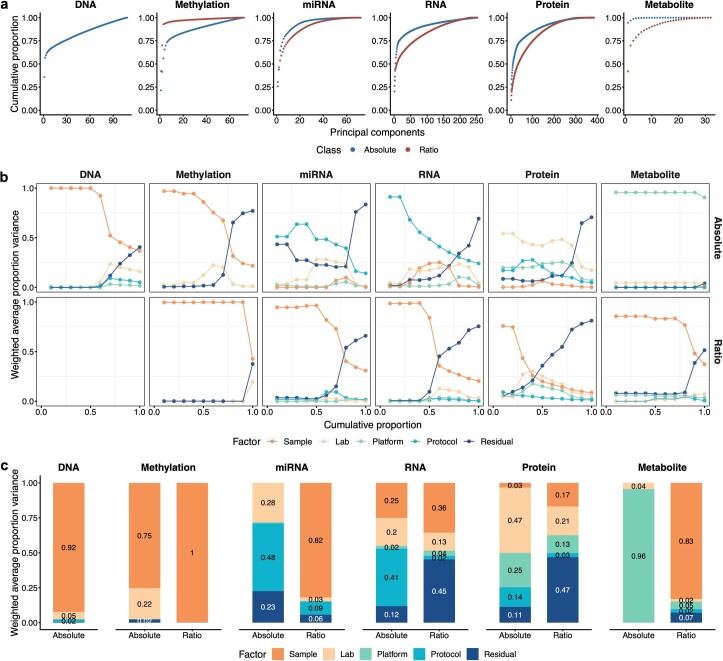
Sources of variability in the Quartet multi-omics datasets at the absolute and ratio levels. **a**, Scatter plots between cumulative proportion and principal components at absolute (Blue) and ratio (Red) levels. **b**, The principal variance component analysis plots measuring the contribution of impact factors to the Quartet multi-omics profiles at absolute (Top) and ratio (Bottom) levels. The impact factors included sample (Orange), lab (Light yellow), platform (Green), protocol (Blue), and residual (Dark blue). The x-axis indicates the cumulative proportion of variance explained from 0.1 to 1 in increments of 0.1, and the y-axis indicates the weighted average proportion variances. **c**, Bar plots of PVCA when the cumulative contribution of variance explained was 60%. The annotated scores were weighted average proportion variances.

We further compared the sources of variability in the Quartet data at the absolute and ratio levels. Technical factors dominated the variability in the absolute data, and the proportional contribution of each factor to the total variability is dependent on omics type (Extended Data Fig. 4). On the contrary, principal-variance component analysis (PVCA) results for the ratio data showed that the biological factor (‘donor/sample’) dominated the data variability in most omics types and its relative contribution over technical factors was markedly higher when compared to the absolute data. The PVCA results clearly demonstrated the effectiveness of feature-by-feature ratio data in removing technical noise present in absolute multi-omics data, enabling the identification of true biological signals (that is, true differences among donors).

**Extended Data Fig. 5:**
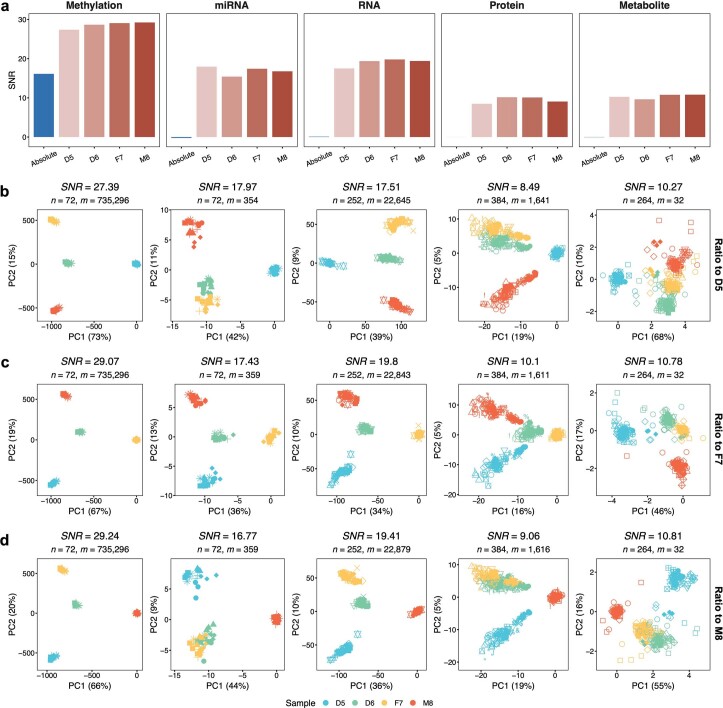
Ratio-based integration enhanced horizontal data integration no matter which sample was chosen as the reference. **a**, Bar plots of Signal-to-Noise Ratio (SNR) of horizontal integration of all batches of methylation, miRNAseq, RNAseq, proteomics, and metabolomics datasets at absolute level (Blue) and ratio level (Red) with the choice of different Quartet samples as the reference sample. **b-d**, PCA plots of horizontal integration of omics datasets at ratio level by scaling to D5 (**b**), F7 (**c**), and M8 (**d**).

The reliability of horizontal integration can also be assessed using the Quartet-based SNR metric. The aforementioned five types of quantitative omics data all showed obvious batch-dominant clustering at absolute expression levels in horizontal integration (Fig. 3d). However, after converting the absolute omics data to a ratio scale relative to the same reference material (D6) within a batch on a feature-by-feature basis, PCA plots showed clear separation of the four types of reference samples (D5, D6, F7 and M8) and the strong batch effects seen at the absolute scale were largely absent (Fig. 3e). We further quantitatively measured the quality of horizontal data integration using the Quartet multi-sample-based SNR as the metric. A method of good quality for horizontal data integration at each omics level would clearly separate the four Quartet sample groups; that is, the inter-sample differences of the Quartet samples should be much larger than the variation among technical replicates of the same sample. As shown in Fig. 3d,e, the SNR values after horizontal integration of datasets for each omics type at the absolute level were all close to zero except for methylation data (Fig. 3d), whereas the SNR values of the integrated datasets were markedly higher at the ratio level for each omics type (Fig. 3e). Notably, these conclusions remain the same if one chooses D5, F7 or M8 instead of D6 as the reference sample (Extended Data Fig. 5), indicating the universal applicability of the ratio-based scaling approach.

In addition, we characterized the impact of the level of batch effects on the SNR for horizontal integration by randomly selecting samples from different batches and using the average of the Jaccard index for batches from the four sample groups as a measure of group-batch balance (see Methods for details). As shown in Fig. 3f, regardless of the level of balance of sample classes across batches, horizontal integration at the ratio level resulted in much better discrimination between sample classes, that is, much higher SNR. However, the corresponding SNR values at the absolute level were all close to zero except for methylation data, whether there was group-batch balance or not. These results clearly demonstrate that quantitative omics profiling data at the ratio level are much more comparable and suitable for horizontal integration than those at the absolute level.

Ratio-based profiling allows for more accurate determination of the subtle differences between any two Quartet samples on a feature-by-feature basis. For all three comparisons (D5–F7, D5–M8 and F7–M8), compared to the log_2_-transformed fold differences in the absolute-based integration data, those for the ratio-based integration data showed a much higher level of agreement (and lower RMSE) with the corresponding reference dataset for each omics type (Extended Data Fig. 3b,c). Furthermore, the level of balance of sample groups across batches was helpful for the accurate detection of DEFs. This was reflected in the negative correlation between RMSE and the level of group-batch balance. It was also clear that a lack of group-batch balance affected absolute data integration much more severely than it did ratio-based data integration, with the former showing a much larger slope than the latter (Extended Data Fig. 3c).

The pervasiveness of batch effects in quantitative analysis techniques at the absolute expression level presents a real challenge for horizontal integration. Our results demonstrate that the conversion of quantitative omics data to a ratio scale relative to a common reference sample (for example, the Quartet D6 sample) can effectively mitigate the detrimental impact of batch effects on sample classification, differential feature identification, etc.

### Improved reliability of cross-omics feature correlations

One advantage of multi-omics studies is the ability to systematically discover cross-omics relationships from multiple interconnected biological layers. The correlation coefficient is one of the simplest ways to estimate the pairwise relevance for two types of omics features, which is the foundation of multi-omics integration for network analysis. In large multi-omics studies, the multi-omics datasets are usually generated in multiple batches, platforms and labs. Vertical integration of multi-omics datasets from various omics types is typically performed after horizontal integration of the same omics type^73^; thus, performance of the final integration is influenced by both horizontal and vertical dimensions. Therefore, we evaluated the reliability of vertical integration using horizontally integrated ratio-based data under different scenarios.

**Fig. 4:**
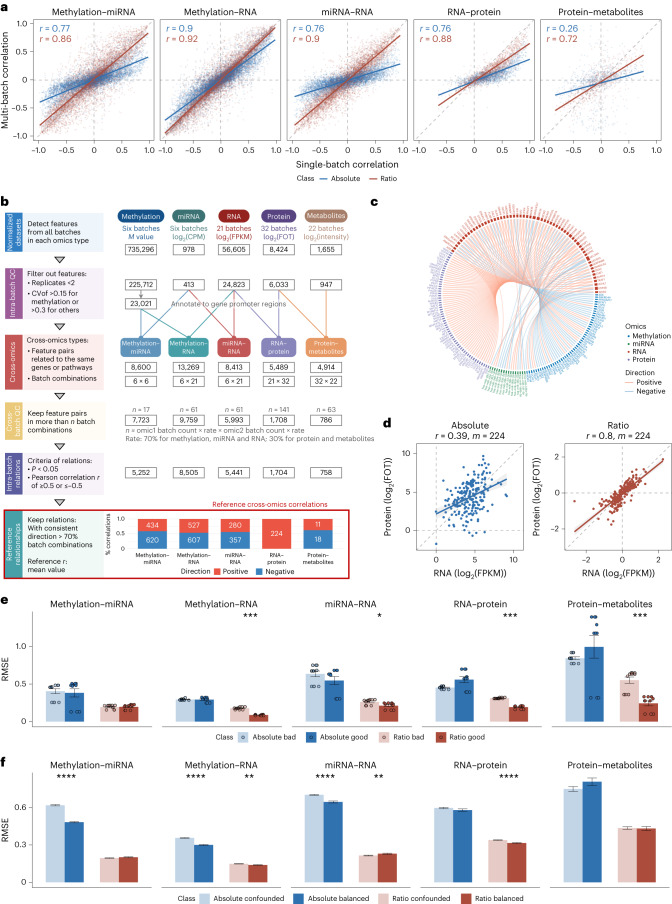
Improved reliability of cross-omics feature correlations. **a**, Scatterplots of the cross-omics feature relationships of intra- and inter-batch (horizontally integrated) data at the absolute level (blue) and ratio level (red). The solid lines represent fitted curves from linear regression along with the Pearson correlation coefficient (*r*). **b**, Workflow for the construction of reference datasets of cross-omics feature relationships according to the following steps: (1) identification of detectable multi-omics features and per-sample normalization; (2) intra-batch QC by filtering out features that are not detectable or have low technical reproducibility; (3) identification of cross-omics feature pairs associated with the same genes or pathways; (4) cross-batch QC by retaining reliable feature pairs identified in a sufficient number of batches; (5) calculating Pearson correlation coefficients for each feature pair in each batch combination and classifying the relationships into positive (*r* ≥ 0.5, *P* < 0.05) and negative (*r* ≤ –0.5, *P* < 0.05) categories; and (6) voting based on the direction of the correlations (negative or positive) to screen the high-confidence cross-omics feature relationships. **c**, Chord plot of the reference dataset of cross-omics feature relationships. Each chord represents a positive (red) or negative (blue) correlation of any two cross-omics features. **d**, Scatterplots of the expression abundance of 224 positively correlated RNA–protein pairs at the absolute level (blue) and ratio level (red). Data were selected from the best quality batch in the RNA-seq and proteomics datasets. *r* denotes the Pearson correlation coefficient, and *m* denotes the number of features. **e**,**f**, Bar plots of RMSE of cross-omics feature relationships identified from different quality datasets (**e**; bad versus good) and different scenarios (**f**; confounded versus balanced) at the absolute level (blue) and ratio level (red) based on the reference datasets. The number of data sampling instances (*n*) used to derive statistics was as follows: bad, *n* = 10; good, *n* = 10; confounded, *n* = 200; balanced, *n* = 100. Data are presented as mean values ± s.d. The *P* values were calculated using unpaired two-tailed Wilcoxon rank-sum tests with false discovery rate (FDR) correction. ****P < 0.0001, ***P < 0.001, **P < 0.01, *P < 0.05; not significant, *P* ≥ 0.05. Specific *P* values are listed in Supplementary Data 1 and 2.

Cross-omics feature relationships calculated on the basis of multiple batches of data integrated at the ratio level (inter-batch) showed much stronger correlations with cross-omics single batches (intra-batch) than those at the absolute level (Fig. 4a). These cross-feature correlations of methylation–miRNA, methylation–RNA, miRNA–RNA, RNA–protein and protein–metabolite types were derived from features of both omics types associated with the same genes, which may more closely follow the principle of the central dogma. In particular, for the relationships between proteins and metabolites, direct integration of multi-batch data at the absolute level could not easily identify true correlations for cross-omics feature pairs.

To evaluate the performance of vertical integration at the feature relationship level, we constructed Quartet cross-omics reference datasets (Supplementary Table 3) using a consensus voting approach, as depicted in Fig. 4b. This reference dataset consisted of the Pearson correlation coefficients between the expression levels of two different types of omics features. By exhaustively enumerating all batch combinations of the above five cross-omics types, feature pairs that exceeded a predetermined threshold of batch combinations were selected for further analysis. The cross-omics relationships were classified as positive (*r* ≥ 0.5, *P* < 0.05) or negative (*r* ≤ –0.5, *P* < 0.05) on the basis of the outcomes of Pearson correlation analysis conducted for each feature pair. Feature pairs demonstrating positive or negative correlations above 70% of all batch combinations were included in the high-confidence dataset, and the mean value for this category (that is, positive or negative) was used as the reference Pearson correlation coefficient.

The reference dataset comprises a comprehensive selection of high-confidence correlation feature pairs, consisting of 1,054 methylation–miRNA pairs, 1,134 methylation–RNA pairs, 637 miRNA–RNA pairs, 224 RNA–protein pairs and 29 protein–metabolite pairs (Fig. 4b). Within this dataset, a subset of 59 genes showed regulation influenced by both methylation and miRNA, alongside a strong positive association with protein (*P* < 0.05, *r* > 0.5), as depicted in Fig. 4c. This finding highlights the intricate interplay between different omics types and offers valuable insights into the coordinated regulation of gene expression.

**Extended Data Fig. 6:**
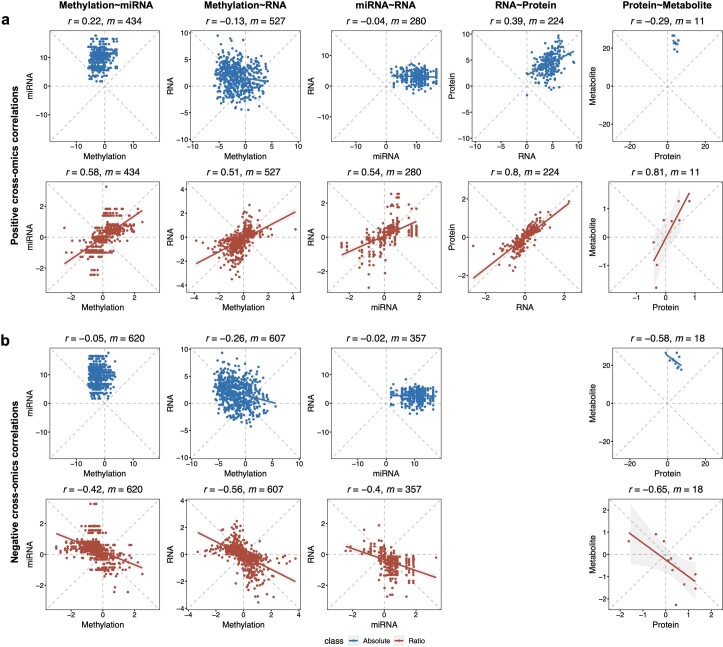
Ratio-based scaling improved the identification of cross-omics feature associations in reference datasets. Scatter plots of the abundance of positively correlated (**a**) and negatively correlated (**b**) cross-omics features in the reference dataset at the absolute (Blue) and ratio (Red) levels. The Pearson correlation coefficients were denoted by *r* and the number of features were denoted by *m*. Data points represent one feature and solid lines indicate fitted lines obtained from linear regression. The shading indicates the 95% confidence intervals.

The principle of the central dogma was well reflected in the Quartet multi-omics data, as it could be seen that the abundance of RNAs was almost exclusively positively correlated with that of proteins in the reference dataset (224 RNA–protein pairs were positively correlated while no RNA–protein pair was negatively correlated). The positive RNA–protein correlations were better identified at the ratio level (*r* = 0.8) than at the absolute level (*r* = 0.39; Fig. 4d). The same phenomenon was demonstrated for other inter-omics associations; that is, ratio-based scaling improved the confidence of the identification of cross-omics feature relationships in the reference datasets (Extended Data Fig. 6).

In large-scale cohort studies involving multi-omics quantitative analyses, issues related to uneven data quality and unbalanced sample groupings across batches often arise^36,37^. Confounded scenarios, characterized by substantial confounding between biological factors and batch effects, are frequently encountered in longitudinal and multicenter cohort studies, presenting challenges in disentangling the influences of biological factors from batch effects. Although balanced scenarios, where samples from the biological group of interest are evenly distributed across batches, represent an ideal situation, they are rarely achievable in practical settings. In this context, we further investigated the performance of the ratio-based approach under both scenarios.

In agreement with Fig. 4a, the concordance of correlation coefficients of cross-omics features with the reference Pearson *r* was higher (as indicated by lower RMSE values) in the horizontally integrated data based on the ratio level than those based on the absolute level (Fig. 4e,f). The ratio-based profiles exhibited lower RMSE values when detecting cross-omics feature relationships from datasets of different quality (as indicated by the SNR values). The performance of identifying cross-omics feature relationships on the basis of ratio-based data is improved when the single-batch dataset is of higher quality (Fig. 4e). Furthermore, in different experimental scenarios, that is, balanced or confounded batch groups, the ratio-based data showed essentially the same good performance, whereas the absolute level was more sensitive to batch effects (Fig. 4f).

### Facilitating vertical integration for sample classification

Another advantage of vertical integration of multi-omics data is the ability to distinguish subtypes of clinical samples with subtle differences that cannot be identified on the basis of a single type of omics data. Therefore, the ability to discover the true biological differences between sample groups is a key metric to measure the performance of multi-omics integration tools and procedures. The multi-sample and multi-omics design of the Quartet Project provides unique resources for assessing the reliability of vertical integration. Here we included six horizontal integration methods for evaluation, that is, ratio-based scaling (Ratio), ComBat^74^, Harmony^75^, RUVg^76^, *z* score and direct integration of the normalized values (Absolute). Five widely accepted vertical integration tools were subsequently used, that is, SNF^5^, iClusterBayes^77^, MOFA+^78^, MCIA^79^ and intNMF^80^, generating 30 combinations of horizontal and vertical integration for performance assessment.

The adjusted Rand index (ARI)^81^ is a widely used QC metric to compare clustering results against external criteria. To quantitatively evaluate the reliability of vertical data integration at the multi-omics level, we used Quartet-based ARI (daughter1–daughter2–father–mother (that is, D5–D6–F7–M8) as four independent sample groups or clusters) as the metric.

**Fig. 5:**
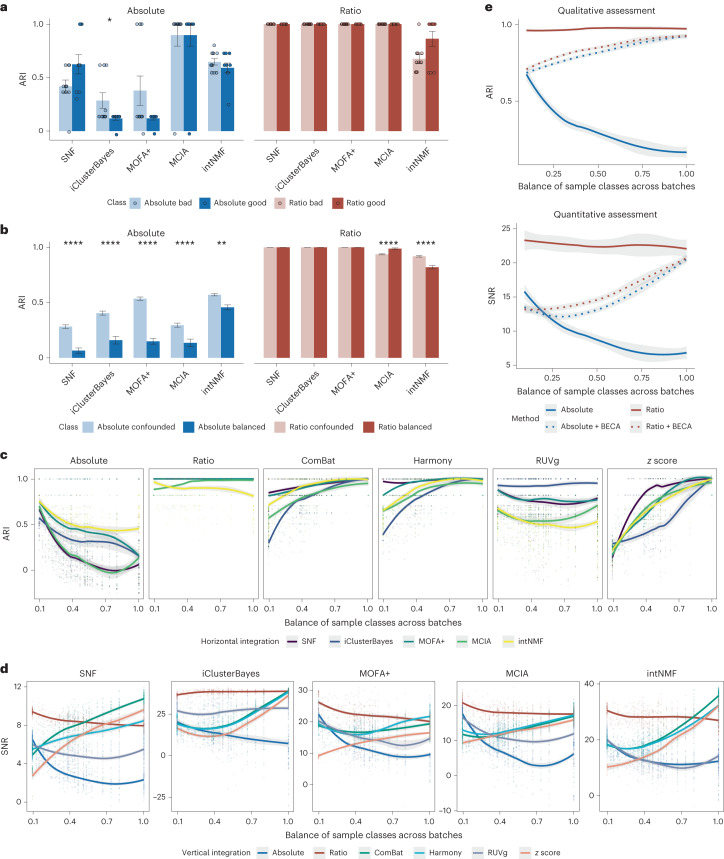
Facilitating vertical integration for sample classification. **a**,**b**, Bar plots of the ARI of vertically integrated multi-omics datasets of different quality (**a**; bad versus good) and different scenarios (**b**; confounded versus balanced) at the absolute level (blue) and ratio level (red) using SNF, iClusterBayes, MOFA+, MCIA and intNMF. The number of data sampling and integration instances (*n*) used to derive statistics was as follows: bad, *n* = 10; good, *n* = 10; confounded, *n* = 200; balanced, *n* = 100. Data are presented as mean values ± s.d. The *P* values were calculated using unpaired two-tailed Wilcoxon rank-sum tests with FDR correction. ****P < 0.0001, **P < 0.01, *P < 0.05; not significant, *P* ≥ 0.05. Specific *P* values are listed in Supplementary Data 3 and 4. **c**, Scatterplots of the degree of sample class-batch balance versus ARI with different data preprocessing methods. **d**, Scatterplots of the degree of sample class-batch balance versus SNR with different data preprocessing methods. SNR was calculated on the basis of a sample-to-sample similarity matrix. **e**, Curves of ARI and SNR with the degree of balance between sample classes and batches at the absolute level (blue, solid line), ratio level (red, solid line), absolute level combined with BECAs (blue, dotted line) and ratio level combined with BECAs (red, dotted line). Each point represents an instance of data sampling and integration. The solid lines correspond to fitted curves obtained from local regression, and the shading indicates the 95% confidence interval around the smoothing.

**Extended Data Fig. 7:**
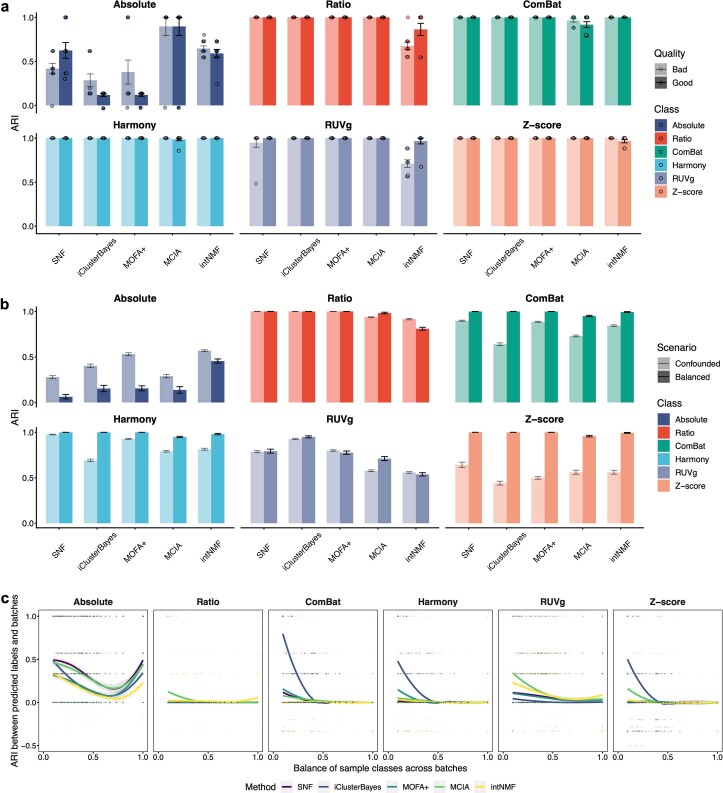
Vertical integration with different data preprocessing methods. **a**,**b**, Bar plots of the Adjusted Rand Index (ARI) of vertically integrated multi-omics datasets of different quality datasets (**a**, Bad vs. Good) and different scenarios (**b**, Confounded vs. Balanced) at absolute level (Blue) and ratio level (Red) using SNF, iClusterBayes, MOFA + , MCIA, and intNMF. Data of each omics type were preprocessed by Absolute (no further processing on the normalized datasets), Ratio, ComBat, Harmony, RUVg, or Z-score for horizontal integration. The number of data sampling and integration instances (n) used to derive statistics were as follows: Bad, n = 10; Good, n = 10; Confounded, n = 200; Balanced, n = 100. Data are presented as mean values ± SD. **c**, Scatter plots between ARI between predicted labels and batches as well as the degree of sample class-batch balance with different data preprocessing methods. Each point represents an instance of data sampling and integration. The solid lines depict local regression fit of the data and shaded regions depict 95% confidence intervals.

Ratio-based scaling data largely outperformed the absolute-level data with a much higher ARI when the same vertical integration algorithm was used (Fig. 5a). Furthermore, there was no significant difference between high- and low-quality groups, regardless of the methods used (Extended Data Fig. 7a). This may be due to the relatively simple classification task and the integration of multi-omics data that effectively improves the discrimination between different samples.

In particular, ratio-based data showed an obvious advantage over the absolute level in confounded scenarios (Fig. 5b and Extended Data Fig. 7b). The vertical integration based on the ratio-level profiles exhibited an ARI close to 1 at different levels of batch-group balance. Most of the popular batch correction methods (ComBat, Harmony and *z* score) showed lower ARI in the confounded scenario, and their performance with all five vertical integration algorithms steadily improved as the degree of batch-group balance increased. RUVg, which is theoretically suitable for processing confounded datasets, had excellent performance in extremely confounded and balanced scenarios. However, it was progressively less effective when the batch and sample groups were confounded. Interestingly, the absolute-level data exhibited a similar pattern of variation as RUVg, with the highest ARI under extreme confounding scenarios. However, it was batch information that was actually distinguished (Fig. 5c and Extended Data Fig. 7c). These results suggest that vertical integration for sample classification based on ratio-based scaling profiles is essentially unaffected by the degree of batch-group balance in the experimental design.

It is worth noting that ARI only qualitatively measures whether clustering results and external criteria have a similar clustering structure and does not indicate the degree of difference between clusters. When the ARI is the same, the biological features of sample groups after vertical integration may still differ. In this context, we extended the idea of SNR to quantitatively evaluate the vertically integrated results to improve the resolution of the assessment of the accuracy of sample classification (see Methods for details). In line with the previous findings, ratio-based scaling resulted in higher SNR values in different scenarios ranging from confounded to balanced, regardless of the vertical integration method used (Fig. 5d).

The ultimate performance of integration was influenced by both the horizontal integration methods and the vertical integration algorithms. For example, regardless of the chosen horizontal integration method, MOFA+ performed better than MCIA in subsequent vertical integration. These results indicate that ratio-based scaling improves the vertical integration of sample clusters through reliable cross-sectional integration.

By conducting a comprehensive comparison with BECAs, we aim to provide a more robust depiction of the effectiveness of direct quantification at the ratio level during data generation. When using ratio quantification directly, the ratio approach consistently produces high ARI values, indicating accurate sample classification, as well as high SNR values, indicating discriminatory power to correctly classify samples, regardless of whether the sample classes are balanced across batches. Furthermore, the additional use of BECAs in conjunction with ratio quantification produces superior outcomes compared to batch correction based on absolute quantification (Fig. 5e). Therefore, it is imperative to incorporate ratio-based profiling at the experimental measurement stage instead of data massage alone (for example, normalization and/or batch effect correction) after data generation.

### Quartet design for genetics-driven ground truth

Multi-omics integration of molecular-level information and phenotypic characteristics holds great promise in advancing understanding of intricate genotype–phenotype relationships. Beyond straightforward differentiation of the four different individuals (daughter1–daughter2–father–mother, or D5–D6–F7–M8), the Quartet monozygotic twin family design offers a unique opportunity as well as a more challenging task of classification into the Quartet family-based groups and three genetically distinct groups (daughters–father–mother, or D–F–M). Here we integrated the multi-omics data of moderate quality (SNR in the range of the top 20% to 80%) including DNA variants, methylation, miRNA, RNA, protein and metabolites. For each vertical integration method, only one batch of data was selected for each omics type to prevent the influence of batch effects during horizontal integration. In addition, we conducted partitioning around medoids (PAM) clustering^82^ for each type of single omics data and calculated ARI as a control to assist in assessing the performance of the vertical integration.

**Fig. 6:**
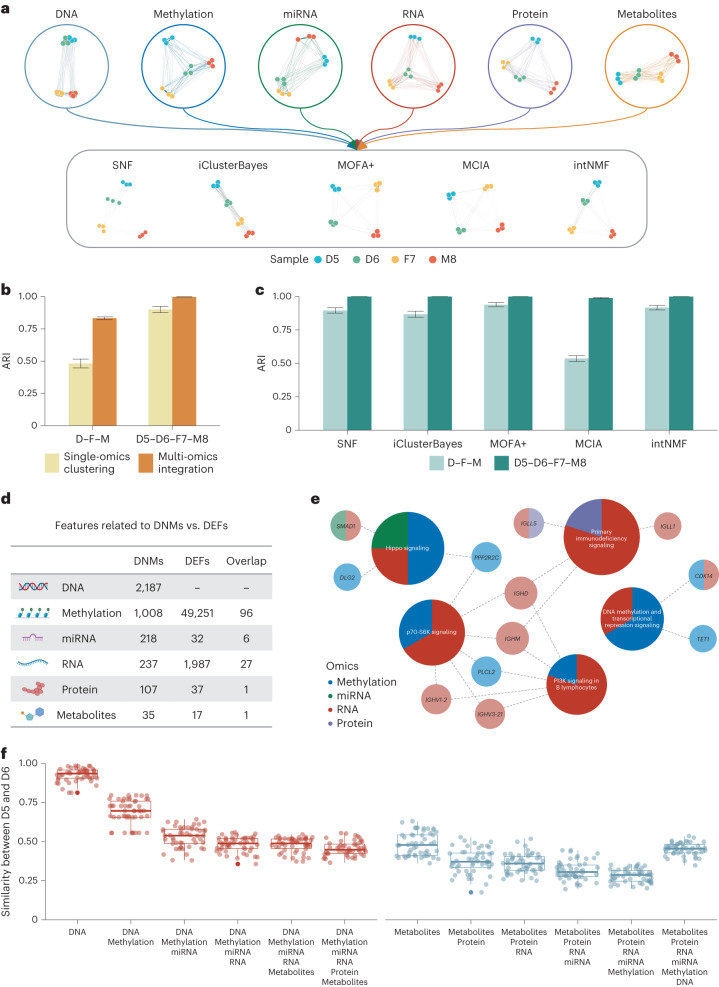
Quartet design for genetics-driven ground truth. **a**, Networks of six types of omics profiling based on the similarity between 12 samples within one batch (top) and sample similarity networks obtained with SNF, iClusterBayes, MOFA+, MCIA and intNMF (bottom), which integrated the six types of multi-omics data. **b**, Bar plots of the ARI when clustering samples into three (D–F–M) or four (D5–D6–F7–M8) groups by single-omics clustering (yellow) versus multi-omics integration (orange). **c**, Bar plots of the ARI for multi-omics data integration using SNF, iClusterBayes, MOFA+, MCIA and intNMF. Light green represents data when the true labels of the samples were set to three clusters (D–F–M), while dark green represents four clusters (D5–D6–F7–M8). In **b**,**c**, data are presented as mean values ± s.d. A total of 60 batches of multi-omics datasets were used for single-omics PAM clustering, on the basis of which 100 cross-omics combinations were used for multi-omics integration with five algorithms. **d**, The number of multi-omics features associated with DNMs, DEFs identified from profiles and their intersections. **e**, Enrichment pathway maps for differential multi-omics features between D5 and D6, that is, the intersection of DNMs and DEFs. Darker colors indicate pathways and lighter colors indicate genes. The percentage of each circle of a specific color corresponds to the proportion of features associated with each omics type. **f**, Box plots of the similarity between D5 and D6 for integration of different types of omics data with 50 iterations. The multi-omics data were integrated starting with DNA (red) and ending with metabolites (gray) by using SNF. The box plots display the distribution of data with the median represented by the line inside the box and the interquartile range represented by the box. Whiskers extend to 1.5× the interquartile range.

The inter-sample similarity networks built using data from a single omics type (top) and multi-omics data integrated using SNF, iClusterBayes, MOFA+, MCIA and intNMF (bottom) are visualized in Fig. 6a. At the DNA level, the samples for the identical twins (D5 and D6) were tightly clustered together owing to their near-identical DNA sequences. On the other hand, these samples showed no clear tendency to cluster together for the five types of quantitative omics data (methylation, miRNA, RNA, protein and metabolites) and could even appear relatively far apart (for example, D6 and F7 appeared closer in miRNA, RNA and protein data). This distinction in clustering tendency between DNA variants and quantitative omics data implies that the classification task (D–F–M) can be used to assess whether a vertical integration approach can reveal the intrinsic, built-in genetic truth in the Quartet family with identical twins.

Vertical integration reduced technical noise and improved sample clustering, as indicated by the fact that the ARIs for both the three clusters (D–F–M) and four clusters (D5–D6–F7–M8) from multi-omics integration were higher than those with direct clustering of single-omics data (Fig. 6b). Nevertheless, there were still differences in performance between the vertical integration algorithms when distinguishing the three sample categories (D–F–M). SNF, iClusterBayes, MOFA+ and intNMF correctly classified the samples into the three Quartet family-based groups (D–F–M), whereas MCIA did not perform well (Fig. 6c). This demonstrates that the integration algorithms could be prioritized by whether they find potential genetic truth (identical twins) behind the four individuals with distinct differences in molecular phenotypic data.

To better decipher what influences D–F–M clustering, we annotated the genomic coordinates of de novo and somatic small variants (abbreviated as DNMs) in addition to directly calculating DEFs for each omics type. The intersection of these indicates highly plausible multi-omics features affected by genomic-level differences between the Quartet identical twins (Fig. 6d). Further enrichment analysis yielded pathways and features with specific molecular insights into the impact of genomic variants on D–F–M clustering (Fig. 6e). Identification of the primary immunodeficiency signaling pathway (*IGHM*, *IGHD*, *IGLL1* and *IGLL5*) indicated potential differences in immune system functions between the cell lines derived from the twins that could affect immunoglobulin synthesis and secretion, likely resulting from the process of immortalization of B cells with Epstein–Barr virus (EBV). The Hippo signaling pathway (*DLG2*, *PPP2R2C* and *SMAD1*) is associated with cell proliferation, polarity and tissue morphology, suggesting that there could be structural and morphological differences between the two cell lines from the twins. The p70-S6K signaling pathway (*IGHM*, *IGHD*, *IGHV1-2*, *IGLV3-21*, *PLCL2* and *PPP2R2C*) is associated with protein synthesis, cell proliferation and metabolic regulation and could potentially account for variations in the culturing status of the two cell lines. The PI3K signaling pathway in B lymphocytes (*IGHM*, *IGHD*, *IGHV1-2*, *IGLV3-21* and *PLCL2*) is specific to these cells and is also associated with protein synthesis, cell proliferation and metabolic regulation. Finally, identification of DNA methylation and transcriptional repression signaling (*CDK14* and *TET1*) suggested that there may be differences in these processes between the twins. Taking these findings together, it is possible that some of the multi-omics differences between the Quartet identical twins at the immune, cellular and metabolic levels are due to genetic variation. Additional differences may be caused by environmental or random factors.

The similarity between the identical twins (D5 and D6) during vertical integration can be quantified to illustrate the impact of adding different layers of omics information on the clustering of the Quartet samples (see Methods for details). As shown in Fig. 6f, the similarity between D5 and D6 decreased both when gradually adding downstream omics data starting with genomics data (left, red) and when integrating upstream omics data starting with metabolomics data (right, blue; except for the eventual addition of DNA). This phenomenon again demonstrates that the genetic relationships between the Quartet identical twins are only reflected at the DNA level, and it also specifies the need to incorporate genomic data when using the three clusters (D–F–M) as a QC metric for vertical integration.

### Best practices for QC using Quartet reference materials

QC comprises procedures to ensure the reliability of multi-omics profiling using defined QC metrics and thresholds to meet the requirements of different research purposes. Large-scale multi-omics studies involve multicenter and long-term measurements for which unified QC metrics and universal integration strategies are needed to ensure quality during data generation and integration. We recommend including the Quartet reference materials (for example, four samples × three replicates) or a similar strategy when profiling each batch of study samples and propose best-practice guidelines for QC and data integration in three aspects, including intra-batch data generation, horizontal integration and vertical integration (Extended Data Table 2).

We have provided both reference dataset-free and reference dataset-based QC metrics to assess the wet-lab proficiency of data generation for the same omics type in terms of the capacity to identify subtle differences between sample groups. Without relying on the reference datasets, the Quartet-based SNR (D5–D6–F7–M8) can be calculated for quality assessment for all types of omics data. SNR calculated on the basis of the four Quartet sample groups was more sensitive when assessing wet-lab proficiency than generic QC metrics based on multiple technical replicates of a single sample (Fig. 2). We also recommend use of the Mendelian concordance rate based on the pedigree of the Quartet family as a QC metric for assessing the quality of genomic data^66^. With the reference datasets, the wet-lab proficiency was assessed by the concordance between the evaluated batch of data and the reference datasets. Precision, recall and F1-score are recommended for qualitative omics (small variants and SVs), and RMSE at the ratio level (scaling to D6) for feature expression and the differential expression between groups (D5–F7, F7–M8 and M8–D5) is recommended for quantitative omics (DNA methylation, transcriptomics, proteomics and metabolomics). In addition, more comprehensive proficiency tests or inter-lab comparisons can be performed by obtaining the relative quality ranking among the cumulative datasets within the Quartet Data Portal^72^.

For horizontal integration of multi-batch data, a paradigm shift from absolute to ratio-based profiling by incorporating common reference materials is essential and improves the reproducibility and resistance to batch effects. QC metrics used in intra-batch data generation can still be used in the quality assessment of horizontal integration. The reliability of further exploratory studies can be ensured as long as the horizontally integrated dataset can still distinguish the different Quartet samples with subtle built-in differences.

Vertical integration can be enhanced by ratio scaling the data on the basis of reference materials. The Quartet multi-omics and multi-sample reference materials provide two types of metrics for QC of vertical integration. The first type, referred to as the ‘built-in truth’, leverages clustering of the Quartet samples through the combined use of ARI_D–F–M_ and ARI_D6–D6–F7–M8_ to synthetically characterize the quality of vertical integration. In addition, the ability to correctly distinguish samples into four clusters (D5, D6, F7 and M8), as measured by ARI_D6–D6–F7–M8_, indicates that the integrated multi-omics data must have the basic ability to differentiate the four different biological samples from technical replicates. On the other hand, the integration algorithm must be able to identify the multi-omics features driven by the built-in genetic truth of the Quartet identical twins, thus separating samples into three clusters (daughters, father and mother) by identifying true cross-omics associations. The second type of metrics focus on the hierarchical relationship across omics features following the principle of the central dogma. RMSE of cross-omics feature relationships calculated on the basis of the high-confidence reference datasets can be used to evaluate the accuracy of the cross-omics feature correlations. These metrics provide insights into the fidelity of vertical integration by assessing the consistency between integrated data and known biological relationships.

## Discussion

We developed suites of publicly available multi-omics reference materials, including matched DNA, RNA, protein and metabolites from immortalized LCLs of four individuals from a Chinese quartet family. We then profiled these reference materials using diverse multi-omics technology platforms in multiple labs across batches with repeated measurements. The reference datasets of measurands characterizing these reference materials at genomic scale were established on the basis of a consensus approach using multiple bioinformatics pipelines and data integration approaches. The Quartet reference materials and the reference datasets can facilitate objective quality assessment of multi-omics profiling by providing two types of metrics for QC of multi-omics data generation and data integration. One relates to the built-in truth clustering of the Quartet samples based on their intrinsic and subtle biological differences, and the other relates to the inherent relationships across omics features following the central dogma (DNA to RNA to protein). The resulting wealth of multi-omics resources has been made publicly available through the Quartet Data Portal (https://chinese-quartet.org/).

Wet-lab proficiency was consistently found to be a more important factor affecting the quality of data generated for each omics type than the choice of a specific technology platform (Fig. 2). Our findings are consistent with what has been reported previously on gene expression profiling with microarrays in MAQC-I^55^ and with RNA-seq in MAQC-III (SEQC)^54^ when the same pair of MAQC reference RNA samples, samples A (a mix of RNA from ten cancer cell lines) and B (a mix of RNA from the brain tissues of 23 donors), were analyzed using a given platform in multiple labs. This observation seems intuitive; however, no adequate solution has been validated or adopted by the scientific community, which has likely contributed to the lack of reproducibility of biomedical research^83^. Our observation highlights the urgent need for highly sensitive proficiency testing and training to improve internal lab proficiency before profiling precious research and clinical samples. To this end, we have established appropriate reference materials and propose sensitive metrics for performance assessment.

The ability to correctly identify molecular phenotypic differences between various groups of samples or clinical subtypes of a disease is a fundamental requirement for any omics technology-based research. Thus, an appropriate performance metric should be taken into account and multiple groups of samples must be included to meet this vital requirement. For each omics type, the Quartet study design included four groups of samples (D5–D6–F7–M8), allowing us to define a universal SNR metric for measuring the performance of any multi-omics technology. We found that the SNR metric was sensitive in identifying low-quality datasets that may otherwise be considered of high quality. For example, reproducibility of repeated measurements (or technical replicates) of the same sample, usually expressed as CV, Pearson correlation coefficient or Jaccard index, is a widely used metric for identifying quality issues in transcriptomics, proteomics and metabolomics data^57,62,84^. However, our study demonstrated the limitations of such single-sample-based metrics. In particular, a high Pearson correlation coefficient between technical replicates from a single sample did not ensure high quality in detecting the intrinsic biological differences between different groups of samples (Supplementary Fig. 2). Under such scenarios, unfortunately, the inter-sample differences between different groups of samples (that is, signal) and the intra-sample differences between technical replicates of the same sample (that is, noise) are at the same level, indicating that the measurement system does not have any differentiating ability. The Quartet multi-sample-based reference material suites and the SNR metric offer indispensable advantages in reliability assessment for each type of omics profiling.

Our results urge a paradigm shift from absolute to ratio-based profiling by incorporating common reference materials in the design and execution of multi-omics studies. A striking finding of our study was that multi-omics profiling data at the absolute level, such as FPKM in transcriptomics, FOT in MS-based proteomics and relative peak area in metabolomics, from a single sample are inherently irreproducible across platforms, labs and batches, leading to ‘batch effects’. Such batch effects, usually confounded with study factors of interest, hinder the discovery of reliable biomarkers either by mistaking batch differences as biological signals or by attenuating biological signals with the incorrect use or overuse of ‘batch effect correction’ methods (see details in an accompanying paper^71^). The presence of batch effects makes the horizontal integration of diverse datasets of the same omics type impossible, as can be seen by the lack of the ability to correctly cluster the Quartet samples (Fig. 3d; with SNR close to zero). Convincingly, by converting absolute profiling data for study samples to ratio scales relative to data from the same reference material (such as D6), the resulting ratio-based profiling data (such as D5/D6) were comparable across different protocols, instruments, labs and batches (Fig. 3e; with SNR much improved) and therefore were defined as quantitative reference datasets (Supplementary Table 2).

**Extended Data Fig. 8:**
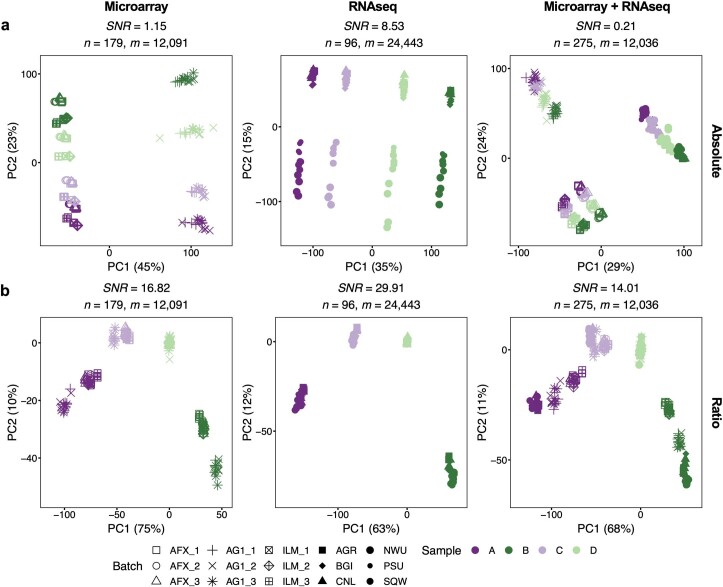
Integration of the microarray and RNAseq datasets of reference RNA samples A, B, C, and D from the MAQC and SEQC1 consortia. PCA plots integrating microarray datasets from MAQC-I (12 batches), RNAseq datasets from SEQC1 (six batches, solid points), and both datasets (18 batches, hollow points) at the absolute (**a**) and relative (**b**) levels by ratio to sample D.

Ratio-based quantitative multi-omics profiling using common reference materials other than the Quartet samples also empowers data integration by substantially removing technical variability in absolute abundance data. We integrated microarray datasets^55^ and RNA-seq datasets^54^ from four well-characterized reference RNA samples, samples A (universal human reference RNA), B (human brain reference RNA), C (a mixture of samples A and B in a 3:1 ratio) and D (a mixture of samples A and B in a 1:3 ratio), from the MAQC/SEQC consortia (Supplementary Table 4). At the absolute abundance level, samples were clustered according to batch and there was a clear distinction between microarray and RNA-seq data and among different microarray platforms (Extended Data Fig. 8a). After converting the measurement data to a ratio scale relative to the reference material within a batch on a feature-by-feature basis, PCA plots showed clear separation of the four types of reference samples (Extended Data Fig. 8b). The marked batch effects seen at the absolute scale largely disappeared, and the SNR values of the integrated datasets were much higher. These results indicate that the advantages of ratio-based profiling are generalizable to sample types other than the Quartet cell lines.

The fact that the ratio-based approach can improve reproducibility was mentioned in our MAQC-II studies on microarrays^85^. However, another study with simulated data appeared to conclude otherwise^37^. Unfortunately, the community is still largely using absolute abundance for quantitative profiling. Our present study systematically demonstrates the impact of ratio-based profiling on the quality of individual types of multi-omics data generated by a wide range of current techniques and on the integration of multi-omics data. More notably, our study generated multi-omics reference materials and datasets with ground truth, which should make the main finding of the need for a paradigm shift from absolute to ratio-based multi-omics profiling convincing for the omics field.

The rich resources from our study and the main findings supporting ratio-based profiling for multi-omics data integration are valuable to the community. First, although the concept of ‘ratio’ has been proposed previously for a single omics type^37,54,55,85^, the community is still largely using absolute abundance for quantitative profiling, and the advantages of ratios over absolute abundance for quantitative profiling have not been fully appreciated or realized. The lack of a convincing study as comprehensive as our current one, which includes well-characterized, publicly available multi-omics reference materials and large amounts of multi-omics data from the same reference materials, might be a reason. Second, we clearly show the advantages of ratio-based measurement for profiling multiple omics types simultaneously. Third, we demonstrate the advantages of ratio-based profiling for the integration of multi-omics data. Finally, and notably, most prior studies have been based on either existing datasets or simulated datasets. By contrast, our study generated multi-omics reference materials and datasets in a systematic and well-thought-out manner to specifically investigate the underlying reasons behind the ‘idiosyncratic’ batch effects^37^ that have been hindering the identification of reliable omics biomarkers for realization of precision medicine.

The large differences in data reproducibility between absolute and ratio-based profiling can be explained, at least in part, by the fundamental principles and assumptions behind data representation for omics measurements. The concentration or abundance (*C*) of an analyte in a sample is important to biomedical research and what a measurement technology intends to provide. In quantitative omics profiling, the absolute instrument readout or intensity (*I*; for example, FPKM, FOT or peak area, regardless of whether per-sample scaling or normalization is applied) is typically used as a surrogate for *C* by assuming that there is a linear and fixed relationship (*f*, or sensitivity) between *I* and *C* under any experimental conditions^86^ such that *I* = *f*(*C*). In reality, however, the relationship *f* can vary because of differences in platform details, reagent lots, lab conditions or operator biases, among other experimental factors, making *I* inherently irreproducible across batches. On the contrary, when a common reference sample (R) is analyzed in parallel with study samples (S) in the same experiment (batch), as a control, the resulting ratio of *I*_S_ to *I*_R_ from each batch will remain reproducible and accurately reflect the ratio of *C*_S_ to *C*_R_. This is because the intensity *I* for the reference and study samples can be represented as *I*_R1_ = *f*_1_(*C*_R_) and *I*_S1_ = *f*_1_(*C*_S_) for batch 1 and *I*_R2_ = *f*_2_(*C*_R_) and *I*_S2_ = *f*_2_(*C*_S_) for batch 2, respectively. Note that *f* remains fixed or comparable for both the reference and study samples being analyzed under the same experiment (batch). Thus, when we divide the intensity *I* of the study sample by that of the reference sample in the same batch, the resulting ratio, *I*_S1_/*I*_R1_ for batch 1 and *I*_S2_/*I*_R2_ for batch 2, will remain the same and equal to *C*_S_/*C*_R_, a constant of biological significance with associated measurement uncertainties. In fact, the lack of reproducibility of absolute gene expression data in microarray^55,87^, RNA-seq^54^ and miRNA-seq^84^ experiments across batches or platforms has been widely documented, as has the increased reproducibility at the ratio scale^54,55,86^. Ironically, mainstream practices still represent omics profiling data on the absolute scale, presumably owing to the lack of readily accessible reference materials as controls, leading to numerous challenges in integrating diverse datasets generated under various experimental conditions. It is gratifying to note that the Olink proteomics platform reports profiling data in ratio scales relative to its control samples (www.olink.com).

Multi-omics profiling is an integrated process bridging genotype and phenotype, and performance validation should be conducted for the entire sample-to-result process. We observed that each component of the data generation and data integration procedures can affect the final results of multi-omics profiling. For each type of omics data generated, full-performance validation and proficiency testing should be conducted to assess whether the measurement system can identify the biological differences inherent between various sample groups, a fundamental goal of multi-omics profiling. Previous studies have mainly focused on performance validation of new technologies^52,60^, but our study revealed that horizontal and vertical data integration across technologies should also be assessed using ground truth-based objective QC metrics. The multi-omics design of the Quartet Project brings a unique dimension to understanding molecular phenomics, allowing us to demonstrate the advantages of multi-omics profiling over any single omics type and to objectively evaluate the pros and cons of various data integration methods in terms of clustering samples according to built-in between-group differences and identifying reliable features with cross-omics relationships obeying the central dogma. The Quartet Project has established a new framework for developing multi-omics reference materials, reference datasets and QC methods for multi-omics studies along with best-practice guidelines for QC and data integration in multi-omics profiling (Extended Data Table 2).

Several limitations and caveats of our study should be pointed out. First, the number of analytes (for example, mRNAs or proteins) expressed in the Quartet reference materials is limited. Each Quartet reference material was derived from a single LCL; thus, genes or proteins not expressed in that LCL are not expected to be detectable in the Quartet reference materials. This is not a serious problem when the purpose is to use the Quartet reference materials for proficiency testing or internal optimization of technology platforms or for training of lab technicians. However, this could become a limitation if the Quartet reference materials were to be used as controls and profiled along with study samples to report ratio-based profiling data, as the denominator for nondetectable features would become zero. In this case, a fudge factor or flooring value can be added to make division possible. Second, the number of analytes with well-defined reference values of differential expression (ratio) between sample pairs is also limited because only large enough ratio values are reproducibly detectable. Third, the long-term stability of the Quartet protein and metabolite reference materials needs to be monitored in terms of both the stability of individual analytes and the stability of the ratio-based reference values. Finally, as is true for any reference materials, replication of the Quartet multi-omics reference materials will require recalibration of the reference datasets and batch-to-batch differences in production and characterization of the reference materials, such as potential genetic drift and variability in quantitative omics features at the methylation, RNA, protein and metabolite levels due to cell culturing, need to be carefully recorded and reported.

In summary, the Chinese Quartet Project provides the international community with rich multi-omics resources, which can serve as a foundation for the research community to evaluate new technologies, labs, assays, products, lab operators and computational algorithms. Large-scale multi-omics studies usually involve complex multicenter and long-term measurements. To ensure the reliability of scientific research results, we highly recommend the use of unified Quartet reference materials or equivalents during generation, analysis and integration of heterogeneous datasets. In particular, the paradigm shift to a ratio-based approach using common references as side-by-side controls, when widely adopted, can fundamentally advance the integration of diverse multi-omics datasets from research and the clinic by making them inherently reproducible and resistant to batch effects, hence increasing the chance of discovering reliable biomarkers to realize precision medicine.

## Methods

### Human participants

This study was approved by the institutional review board of the School of Life Sciences, Fudan University (BE2050). It was conducted under the principles of the Declaration of Helsinki. Four healthy volunteers from a family quartet, as part of the Taizhou Longitudinal Study in Taizhou, Jiangsu, China, were enrolled and their peripheral blood was collected to establish immortalized LCLs. All four donors signed informed consent forms.

### Establishment of the Quartet LCLs

We adopted the widely used protocol in which EBV is used to establish immortalized LCLs^88^. Peripheral blood mononuclear cells were isolated using a lymphocyte separation solution (Ficoll). Naive B cells were sorted by EasySep Human Naive B Cell Enrichment Kit (STEMCELL, 19254) and infected with EBV by centrifugation at 400*g* for 1 h. After incubation, successfully infected and immortalized cells were propagated in culture medium.

### Cell culture

The Quartet LCLs were cultured in RPMI 1640 supplemented with 2 mM l-glutamine, 10% heat-inactivated FBS and 1% penicillin–streptomycin at 37 °C with 5% CO_2_. The cells were passaged every 72 h at a 1:4 split ratio.

### Preparation of the first batch of DNA reference materials

To obtain the first batch of DNA reference materials (lot no. 20160806), 2 × 10^9^ cells were collected simultaneously for each cell line. Specifically, the cells grew in suspension and were centrifuged at 300*g* for 5 min to obtain cell pellets. The cell pellets were then washed twice with cold PBS.

The DNA reference materials were isolated using the DNA with Blood & Cell Culture DNA Maxi Kit (Qiagen) according to the manufacturer’s instructions, divided into 1,000 aliquots for each of the Quartet members and then labeled as Quartet_DNA_D5_20160806, Quartet_DNA_D6_20160806, Quartet_DNA_F7_20160806 or Quartet_DNA_M8_20160806. A single vial contains approximately 10 μg of genomic DNA (220 ng µl^–1^, 50 µl) in TE buffer (10 mM Tris pH 8.0, 1 mM EDTA, pH 8.0).

DNA integrity and long-term stability were evaluated with the Agilent 2200 TapeStation system (Agilent Technologies). Concentrations were determined by NanoDrop ND-2000 spectrophotometer (Thermo Fisher Scientific).

### Preparation of multi-omics reference materials

To obtain the second batch of multi-omics reference materials (lot no. 20171028), 1 × 10^10^ cells were collected for each cell line.

Of these, 2 × 10^9^ cells were used to prepare the second batch of DNA reference materials (lot no. 20171028) with the same method described above for the first batch of DNA. The second batch of DNA reference materials was stocked in 1,000 vials (220 ng µl^–1^, 50 µl) and labeled as Quartet_DNA_D5_20171028, Quartet_DNA_D6_20171028, Quartet_DNA_F7_20171028 and Quartet_DNA_M8_20171028. DNA QC and monitoring of stability were conducted using the same methods described above.

Additionally, 2 × 10^9^ cells pretreated with TRIzol reagent were used to prepare RNA reference materials using the RNeasy Maxi kit (Qiagen) according to the manufacturer’s instructions. The extracted RNA was divided into 1,000 aliquots for the quartet members and labeled as Quartet_RNA_D5_20171028, Quartet_RNA_D6_20171028, Quartet_RNA_F7_20171028 and Quartet_RNA_M8_20171028. A single vial contains approximately 5 μg of RNA in water (520 ng µl^–1^, 10 µl). RNA integrity and long-term stability were assessed with a 2100 Bioanalyzer using RNA 6000 Nano chips (Agilent Technologies) and a Qsep 100 system (BiOptic). Concentrations were determined by NanoDrop ND-2000 spectrophotometer (Thermo Fisher Scientific).

Cell pellets of 2 × 10^9^ cells were used to prepare protein reference materials. Two batches of peptides were prepared separately at Fudan University (on 6 November 2017) and Novogene (on 16 June 2020), China. In brief, cells were lysed in 8 M urea lysis buffer supplemented with protease inhibitors. The extracted proteins were then digested with trypsin overnight at 37 °C. The resulting peptides were divided into 1,000 aliquots and dried under vacuum for each batch of peptide reference materials. Four chemically synthesized peptides with ^13^C- and ^15^N-labeled valine at fixed weight ratios were spiked into the second batch of the reference protein materials (lot no. 20200616) as external controls. The spiked peptides are YILAGVENSK (1:1,000), ADVTPADFSEWSK (1:3,000), DGLDAASYYAPVR (1:9,000) and DSPSAPVNVTVR (1:27,000).

Cell pellets of 1 × 10^9^ cells were used to prepare metabolite reference materials. In brief, cells were extracted using a solution with a 6:1 ratio of methanol to water. Eleven xenobiotics were spiked in at a known amount in each vial as external controls. These included indoleacetic acid (25 pmol), taurocholic acid (1 pmol), glycocholic acid (5 pmol), cholic acid (25 pmol), tauroursodeoxycholic acid (2.5 pmol), taurodeoxycholic acid (7.5 pmol), glycoursodeoxycholic acid (1 pmol), glycodeoxycholic acid (0.5 pmol), ursodeoxycholic acid (25 pmol), deoxycholic acid (50 pmol) and sulfadimethoxine (5 pmol). The cell extracts were divided into 1,000 vials and then dried under vacuum (Labconco) to obtain the cell extracts as metabolomics reference materials. Each vial contains dried cell extract from approximately 10^6^ cells. Stability was monitored by P300 targeted metabolomics using a UPLC–MS/MS system at the Human Metabolomics Institute (Shenzhen, China).

### Whole-genome short-read sequencing data

#### Data generation

To evaluate the intra-lab performance of whole-genome short-read sequencing, three replicates for each of the Quartet DNA samples were sequenced in a fixed order (D5_1, D6_1, F7_1, M8_1, D5_2, D6_2, F7_2, M8_2, D5_3, D6_3, F7_3 and M8_3). A total of 108 libraries from six labs with either a PCR or PCR-free protocol were used in this study. The libraries were sequenced on short-read platforms, including Illumina HiSeq XTen, Illumina NovaSeq, MGI MGISEQ-2000 and MGI DNBSEQ-T7. In paired-end mode, the sequencing depth was at least 30×. More information is detailed in the accompanying paper on DNA^66^.

#### Short-read sequencing read mapping and small variant calling

Read sequences were mapped to GRCh38 (https://gdc.cancer.gov/about-data/gdc-data-processing/gdc-reference-files). Sentieon v2018.08.01 (https://www.sentieon.com/) was used to convert raw fastq files to GVCF files. The workflow included read mapping with BWA-MEM and duplicate removal, indel realignment, base quality score recalibration and variant calling with HaplotyperCaller in GVCF mode. We used default settings for all the processes.

#### Feature encoding for small variants

To perform vertical integration with other quantitative omics data, we used an encoding scheme for the genotypes of SNVs. For each genomic locus, we counted all alleles occurring in a total of 108 samples from nine batches and then encoded them. Heterozygotes that were consistent with the reference genome were encoded as 0, and the others were encoded as 1. We used chromosome 1 to represent the whole genome for the analysis.

### Whole-genome long-read sequencing data

#### Data generation

We evaluated the performance of SV detection using different data analysis pipelines, without considering the technical variation from library preparation. A total of 12 libraries from three long-read sequencing platforms were generated for the Quartet DNA reference materials (one replicate for each sample). The long-read sequencing platforms used were Oxford Nanopore PromethION (~100×), PacBio Sequel (~100×) and PacBio Sequel II (~30×).

#### Long-read sequencing read mapping and SV calling

Reads were mapped to GRCh38 (GCA000001405.15) from the UCSC Genome Brower (http://hgdownload.soe.ucsc.edu/goldenPath/hg38/chromosomes/). Three mappers (NGMLR, minimap2 and pbmm2) and five callers (cuteSV, NanoSV, Sniffles, pbsv and SVIM) were used to call SVs.

### DNA methylation data

#### Data generation

To evaluate the intra-lab performance of DNA methylation analysis, three replicates for each of the Quartet sample groups were assayed in a fixed order (D5_1, D6_1, F7_1, M8_1, D5_2, D6_2, F7_2, M8_2, D5_3, D6_3, F7_3 and M8_3). Methylation data from 72 DNA samples generated by two labs using Illumina Infinium MethylationEPIC v1.0 BeadChip (850k) were used in this study.

#### Preprocessing of methylation data

Raw idat files were processed using the R packages ChAMP (v2.20.1)^89^ and minfi (v1.36.0)^90^. The single-sample Noob (ssNoob) method^91,92^ was used to correct for background fluorescence and dye bias. Next, samples with a proportion of failed probes (probe detection *P* > 0.01) above 0.1 were discarded. Probes that failed in more than 10% of the remaining samples were removed. Probes with <3 beads in at least 5% of samples were also removed. All non-CpG probes, SNP-related probes, multi-hit probes, and probes located on chromosomes X and Y were filtered out. After preprocessing, the methylation dataset contained 735,296 probes. Finally, the corrected methylated and unmethylated signals were used to calculate *M* values and *β* values. In this process, the offset was set to 100 and the *β* threshold was set to 0.001.

### Whole-transcriptome sequencing data

#### Data generation

To evaluate the intra-lab performance of whole-transcriptome sequencing, three replicates for each of the Quartet sample groups were sequenced in a fixed order (D5_1, D6_1, F7_1, M8_1, D5_2, D6_2, F7_2, M8_2, D5_3, D6_3, F7_3 and M8_3). A total of 252 libraries from eight labs with either a poly(A) selection or rRNA removal protocol were used in this study. On average, 100 million read pairs per replicate were sequenced on the Illumina NovaSeq or MGI DNBSEQ-T7 platform. More information is provided in the accompanying paper on RNA^67^.

#### Alignment and RNA quantification

HISAT2 v2.1 was used for read alignment to GRCh38 (version GRCh38_snp_tran; https://genome-idx.s3.amazonaws.com/hisat/grch38_snptran.tar.gz)^93^. SAMtools v1.3.1 was used to sort and convert SAM to BAM format^94^. StringTie v1.3.4 was used for gene quantification with Ensembl reference annotation (Homo_sapiens.GRCh38.93.gtf)^95^. Ballgown v2.14.1 and prepDE.py (https://ccb.jhu.edu/software/stringtie/dl/prepDE.py) were used to produce a gene expression matrix in FPKM for downstream analysis.

### miRNA-seq data

#### Data generation

To evaluate the intra-lab performance of miRNA-seq, three replicates for each of the Quartet sample groups were sequenced in a fixed order (D5_1, D6_1, F7_1, M8_1, D5_2, D6_2, F7_2, M8_2, D5_3, D6_3, F7_3 and M8_3). A total of 72 libraries from four labs using five library kits (NEBNext, NEXTFLEX, TruSeq, Vazyme and QIAseq) were used in this study. The Illumina NovaSeq or HiSeq 2500 platform was used to generate the miRNA-seq data.

#### Alignment and miRNA quantification

An extracellular RNA processing toolkit (exceRpt) was used to preprocess miRNA-seq^96^ data. Raw reads were aligned to the hg38 genome and the transcriptome in exceRptDB. CPM quantification of miRNA was extracted for downstream analysis.

### Mass spectrometry-based proteomics data

#### Data generation

With the first batch of peptide reference materials, 312 libraries based on the LC–MS system were generated under a DDA mode. Samples were analyzed in a random order for each dataset, which contained three technical replicates for each of the four biological samples (D5, D6, F7 and M8). Mass spectrometers from three platforms were used: (1) the Q Exactive hybrid quadrupole–Orbitrap series (Q Exactive, Q Exactive Plus, Q Exactive HF and Q Exactive HF-X), Orbitrap Fusion Tribrid series (Fusion and Fusion Lumos) and Orbitrap Exploris 480 (all from Thermo Fisher Scientific); (2) Triple-TOF 6600 (from Sciex); and (3) timsTOF Pro (from Bruker Daltonics).

The second batch of peptide reference materials were analyzed in a fixed order (D5_1, D6_1, F7_1, M8_1, D5_2, D6_2, F7_2, M8_2, D5_3, D6_3, F7_3 and M8_3) on Q Exactive, Q Exactive HF, Q Exactive HF-X and Orbitrap Fusion Lumos instruments, generating 36 libraries based on DDA mode and 36 libraries based on DIA mode. All parameters were set according to the requirements of the manufacturers. More information is provided in the accompanying paper on protein^68^.

#### Peptide identification and protein quantification

Raw MS files generated using the first batch of peptide reference materials were searched against the NCBI human RefSeq protein database (updated on 7 April 2013, 32,015 entries) using Firmiana 1.0 enabled with Mascot 2.3 (Matrix Science)^97^. Raw MS files generated with the second batch of peptide reference materials were searched against UniProt (http://www.uniprot.org; release-2021_04), using in-house pipelines from different labs (MaxQuant 1.5.3.17, Spectronaut 14.4, mProphet or Proteome Discoverer 2.2). Fixed modifications included carbamidomethylation (cysteine), and variable modifications included oxidation (methionine) and acetylation (protein N terminus). Proteins with at least one unique peptide with 1% FDR at the peptide level and a Mascot ion score greater than 20 were selected for further analysis. FOT values were used for downstream analysis. FOT was defined as a protein’s iBAQ divided by the total iBAQ for all the proteins identified in one sample. The FOT value was multiplied by 10^5^ for ease of presentation.

### Mass spectrometry-based metabolomics data

#### Data generation

To evaluate the intra-lab performance of MS-based metabolomics, three replicates for each of the Quartet sample groups were profiled in a fixed order (D5_1, D6_1, F7_1, M8_1, D5_2, D6_2, F7_2, M8_2, D5_3, D6_3, F7_3 and M8_3). Dried cell extracts were reconstituted in mobile phase in each lab, and a total of 264 libraries were generated from six labs. Nontargeted metabolomics datasets were generated using AB Sciex Triple TOF6600 and Thermo Scientific Q Exactive mass spectrometer systems in three different labs. Targeted metabolomics datasets were generated using Waters Xevo TQ-S, AB Sciex QTRAP 5500 and AB Sciex QTRAP 6500+ mass spectrometers in four labs. More information is provided in the accompanying paper on metabolites^69^.

#### Compound identification and metabolite quantification

Raw data were extracted and underwent peak identification and QC processing using the in-house methods in each lab. Compound identification was conducted using an in-house library on the basis of the retention time/index (RI), mass-to-charge (*m*/*z*) ratio and MS spectral data for each metabolite. Metabolite quantification was conducted using area under the curve or the concentration calculated by calibration curve using standards for each metabolite. All expression tables for metabolomics data were log_2_ transformed.

### Microarray and RNA-seq datasets of reference RNA samples A–D from the MAQC and SEQC consortia

The microarray datasets^55^ and RNA-seq datasets^54^ of four well-characterized reference RNA samples, samples A (universal human reference RNA), B (human brain reference RNA), C (a mixture of samples A and B at a 3:1 ratio) and D (a mixture of samples A and B at a 1:3 ratio), were used to confirm the generalizability of the ratio-based profiling approach. These two datasets spanning 8 years were generated on different platforms across different labs.

Microarray data in the MAQC-I study were generated on three platforms, including Affymetrix, Agilent Technologies (for two colors) and Illumina platforms. Each platform provider selected three sites for testing, and five replicates of each sample were processed at each site. For the RNA-seq data from the SEQC1 study, we selected sequencing data from three sites for the Illumina and Life Technologies platforms, with four replicates of each sample randomly selected from each site. A total of 179 replicates of microarray data and 96 replicates of RNA-seq data were included in this study.

### Data preprocessing

In the analysis of this study, the methylation microarray data were converted to *M* values, miRNA data were normalized to log_2_(CPM), RNA data were normalized to log_2_(FPKM), proteomics data were normalized to log_2_(FOT) and metabolomics data were log_2_ transformed according to the quantitative intensity.

We used different strategies to handle missing values for different omics types when the analysis involved multiple batches of data. For methylation data, features with missing values were removed. For other omics data types, a feature was retained when it was detected in more than 90% of the samples. For miRNA-seq and RNA-seq data, a flooring value of 0.01 was added to each gene’s FPKM or CPM value before log_2_ transformation. For proteomics and metabolomics data, we used the estim_ncpPCA and imputePCA functions of the missMDA v1.18 package^98^ to fill in missing values.

### PCA

PCA was performed using the prcomp function in R statistical packages (v4.0.5). PCA was conducted on single or multiple batches of data from a single omics type. In assessing the quality of single-batch data, we used data from 12 samples (4 donors with 3 replicates per donor) after removing features containing null values and performing normalization. The results can be found in Fig. 2d and Supplementary Figs. 1 and 2.

For multi-batch data quality assessment, we first filtered out features that were null in more than 10% of the samples and then performed imputation as described in the ‘Data preprocessing’ section. PCA was calculated on the basis of the horizontally integrated data, and the corresponding results are presented in Fig. 3d–f and Extended Data Fig. 5.

### PVCA

PVCA was used to measure the contribution of impact factors to the Quartet multi-omics profiles. PVCA uses two statistical methods, that is, PCA and variance component analysis. In addition to biological sample type (donor), technical factors taken into account included lab, platform and protocol. We performed PCA and then estimated the effects of each known factor using the lme4 v1.1-29 package^99^. The residual accounts for variability from other sources or factors not included.

### Differential expression analysis

For each omics type, we identified DEFs in three types of comparisons, that is, D5 versus F7, D5 versus M8 and F7 versus M8, where D6 was used as the common reference (denominator in ratio calculation). Although the four samples were from a family of parents and identical twins, the replicated observations made on the samples were all independent of each other for the quantitative multi-omics characteristics. For the identical distribution problem, the small sample size (*n* = 3) made it impossible to determine precisely whether the two sets of data under comparison were from the same distribution. In this case, direct use of a nonparametric test (for example, the Wilcoxon test) is not an appropriate choice^100^. Therefore, we used a method that has been validated in a series of studies by the MAQC consortium to detect DEFs^47,54,55^.

According to recommendations from the MAQC/SEQC consortia^55,101^, a nonstringent *t*-test *P*-value cutoff with fold-change ranking could be used to identify differentially expressed genes. In this study, we assumed unequal variance and used Welsh’s modification for the degrees of freedom. A feature was identified as differentially expressed when it satisfied the criteria of *P* < 0.05 and log_2_(fold change) of ≥0.5 or ≤–0.5 for miRNA, RNA, protein and metabolite profiling or *P* < 0.05 and log_2_(fold change) of ≥2 or ≤–2 for methylation *M* values. The DEFs were further classified as up- or downregulated on the basis of the positive or negative sign of the log_2_(fold change).

### Workflow for construction of reference datasets

In the analysis of DEFs and cross-omics relationships, intra-batch QC was performed to minimize the influence of technical noise in the voting process. For each sample group, features that were not detected in more than one technical replicate or that had large variability (CV of >0.15 for methylation and >0.3 for other omics types) were excluded. Subsequently, we constructed reference datasets of DEFs and cross-omics feature relationships with the consensus voting approach described below.

#### Reference datasets for DEFs

Cross-batch QC was performed following the previous intra-batch QC. Features retained in more than a certain percentage of batches (70% for methylation, miRNA and RNA; 30% for protein and metabolites) were kept for the subsequent differential expression analysis.

For each omics type, we analyzed the DEFs between D5 and F7 (D5–F7), D5 and M8 (D5–M8) and F7 and M8 (F7–M8) within each batch using the method described in the ‘Differential expression analysis’ section.

After identifying DEFs from each batch, we kept the DEFs present in more than 70% of batches with consistent regulatory directionality (up or down). Finally, we calculated the mean log_2_(fold change) values for all the retained intra-batch DEFs as reference values.

#### Reference datasets for cross-omics feature relationships

The reference datasets contained cross-omics feature relationships between methylation and miRNA, methylation and RNA, RNA and miRNA, RNA and protein, and protein and metabolites. We first performed feature selection to better identify biologically meaningful correlations by annotating cross-omics features to the same genes.

For methylation probes, we converted the features from the level of probes to genes by taking the mean value in the promoter region (TSS200 or TSS1500) to characterize the methylation level. For the other omics types, we did not perform transformation of feature values but simply searched for associated genes. RNA profiles were associated with gene names through Ensembl ID. Target genes associated with specific miRNAs in all of miRDB^102^ (prediction score of ≥80), miRTarBase^103^ (support type of ‘Functional MTI’) and TargetScan^104^ were considered as plausible. The proteomics profiles were characterized at the level of gene names. Metabolites were associated with genes in the same pathway on the basis of the HMDB database^105^.

Subsequently, we exhaustively enumerated all batch combinations of the above five cross-omics types and conducted cross-batch QC. Associated feature pairs retained in more than a certain number of batch combinations were used for subsequent correlation analysis. This threshold was determined by the product of the respective batch and rate (70% for methylation, miRNA and RNA; 30% for protein and metabolites) of the two types of omics data being compared.

Next, we calculated the Pearson correlation coefficient for each feature pair in each batch combination of the five cross-omics types. According to the results of Pearson correlation analysis, the cross-omics relationships were classified as positive (*r* ≥ 0.5, *P* < 0.05), negative (*r* ≤ –0.5, *P* < 0.05) or none (*P* ≥ 0.05).

Finally, we preserved the cross-omics relationships with a category that accounted for more than 70% as high-confidence relationships. The reference Pearson correlation coefficients were the mean value of the retained data.

### Performance metrics

#### ARI

ARI is a widely used QC metric to compare clustering results against external criteria^81^. It measures the similarity of the true labels and the clustering labels while ignoring permutations with chance normalization, which means that random assignments will have an ARI score close to zero. ARI is in the range of –1 to 1, with 1 corresponding to perfect clustering. ARI is calculated on the basis of RI as follows.

$${\textrm{ARI}}=\frac{{{\textrm{RI}}}+{{\textrm{expected}}}\left({{\textrm{RI}}}\right)}{\max \left({{\textrm{RI}}}\right)-{{\textrm{expected}}}\left({{\textrm{RI}}}\right)}$$

#### RMSE

RMSE, the standard deviation of the residuals (prediction errors), is a widely used statistic in bioinformatics and machine learning. In this study, we used RMSE to measure the consistency of DEFs detected from a dataset for a given pair of samples with the reference DEFs, or ‘RMSE of DEFs’. Reference DEFs were integrated by consensus voting the intra-batch results, and the reference difference was defined as the mean value of log_2_(fold change) for high-confidence batches. RMSE is computed using the following equation:

$${{\textrm{RMSE}}}=\sqrt{\frac{\mathop{\sum }\limits_{i=1}^{N}{\left({x}_{i}-{\hat{x}}_{i}\right)}^{2}}{N}}$$

where *N* is the total number of features considered for evaluation, ${x}_{i}-{\hat{x}}_{i}$ is the error, ${\hat{x}}_{i}$ is the log_2_(fold change) after horizontal integration and *x*_*i*_ is the log_2_(fold change) of the corresponding feature in the reference dataset.

#### SNR

SNR is a parameter based on the Quartet study design for discriminating different types of reference samples. On the basis of PCA, SNR is defined as the ratio of the average distance among different samples (for example, D5_1 versus D6_1) to the average distance among technical replicates (for example, D5_1 versus D5_2). SNR is calculated as follows:

$${\rm{SNR}}=10\times {\log }_{10}\left(\frac{m\times \displaystyle\binom{n}{2}}{\displaystyle\binom{m}{2}\times n\times n}\times \frac{\mathop{\sum }\limits_{x=1}^{m}\mathop{\sum }\limits_{y=x+1}^{m}\mathop{\sum }\limits_{i=1}^{n}\mathop{\sum }\limits_{j=1}^{n}\mathop{\sum }\limits_{p=1}^{2}{W}_{p}{({\rm{PC}}_{p,i,x}-{\rm{PC}}_{p,\,j,y})}^{2}}{\mathop{\sum }\limits_{x=1}^{m}\mathop{\sum }\limits_{i=1}^{n}\mathop{\sum }\limits_{j=i+1}^{n}\mathop{\sum }\limits_{p=1}^{2}{W}_{p}{({\rm{PC}}_{p,i,x}-{\rm{PC}}_{p,\,j,x})}^{2}}\right)$$

where *m* is the number of sample groups and *n* is the number of replicates in each sample group. *W*_*p*_ represents the *p*th PC of variance. PC_*p*,*i*,*x*_, PC_*p*,*j*,*x*_ and PC_*p*,*j*,*y*_ represent the *p*th component values of replicate *i* and replicate *j* in sample group *x* or sample group *y*.

The SNR metric was also used for the assessment of clustering accuracy for vertical integration. In this paper, SNR was calculated on the basis of the sample-to-sample similarity matrix output by integration tools, as follows:

$${\rm{SNR}}=10\times {\log }_{10}\left(\frac{\displaystyle\binom{m}{2}\times n\times n}{m\times \displaystyle\binom{n}{2}}\times \frac{\mathop{\sum }\limits_{x=1}^{m}\mathop{\sum }\limits_{i=1}^{n}\mathop{\sum }\limits_{j=i+1}^{n}{({S}_{i,x}-{S}_{j,x})}^{2}}{\mathop{\sum }\limits_{x=1}^{m}\mathop{\sum }\limits_{y=x+1}^{m}\mathop{\sum }\limits_{i=1}^{n}\mathop{\sum }\limits_{j=1}^{n}{({S}_{i,x}-{S}_{j,y})}^{2}}\right)$$

where *m* is the number of sample groups and *n* is the number of replicates in each sample group. S_*i*,*x*_, *S*_*j*__,*x*_ and *S*_*j*__,*y*_ represent the similarity of replicate *i* and replicate *j* in sample group *x* or sample group *y*.

### Balance of sample classes between batches

To evaluate the effect of the level of balance between the sample classes across batches on the horizontal and vertical integration tasks, we used the Jaccard index to represent the level of balance. The Jaccard index is a common statistic used to gauge the similarity and diversity of two sets. For data analysis involving multiple batches, we recorded information on the batches in which the samples of D5, D6, F7 and M8 were located. Further, we calculated the Jaccard index between batches of D5–D6, D5–F7, D5–M8, D6–F7, D6–M8 and F7–M8. Finally, the mean value of the above six Jaccard indexes represented the sample class-batch balance.

### Datasets used in horizontal and vertical integration

#### Randomly sampled quantitative multi-omics datasets

For the multi-batch dataset for each quantitative omics type, we randomly selected an equal subset of all technical replicates from D5, D6, F7 and M8. This number of replicates was greater than three and less than the maximum number for the given omics type. The constituted datasets were used to perform calculation of SNR (Fig. 3f) and for the identification of DEFs (Extended Data Fig. 3a,b).

#### Quantitative multi-omics datasets of different quality (good versus bad)

For each type of quantitative omics profiling (methylation, miRNA, RNA, protein and metabolites), we defined the quality of a batch on the basis of the SNR values. Higher SNR values indicate better data quality, whereas lower SNR values indicate lower data quality. We ranked all batches for each omics type on the basis of their SNR values and classified the top three as good-quality batches (labeled G1, G2 and G3) and the bottom three as bad-quality batches (labeled B1, B2 and B3). Next, we exhausted all combinations of batches from each category, including three combinations of any two batches and one combination of three batches. These datasets were used for cross-omics feature relationship identification (Fig. 4e) and multi-omics data integration for sample classification (Fig. 5a).

#### Quantitative multi-omics datasets under different scenarios (balanced versus confounded)

First, we randomly selected six batches for each type of quantitative omics profiling (methylation, miRNA, RNA, protein and metabolites), resulting in 18 replicates per sample (D5, D6, F7 and M8). This was done because there were only six batches of data for methylation and miRNA. Second, we sorted the column names of the above data by batch, sample and replicate. Next, we constructed balanced and confounded datasets using different sampling methods. To generate a balanced dataset, we randomly selected four unique numbers from 1 to 18 and extracted the corresponding ranked data for samples D5, D6, F7 and M8. To generate a confounded dataset, we drew data from each of the four samples and randomly selected four unique numbers from 1 to 18 to extract the corresponding ranked data. A total of 300 sampled datasets were generated for the data integration analysis. These datasets were applied for cross-omics feature relationship identification (Fig. 4f) and multi-omics data integration for sample classification (Fig. 5b).

#### Genomics and quantitative multi-omics datasets with moderate quality

In the analysis in Fig. 6, the datasets of small variants were added to the integration task. Before vertical integration, we filtered out batches of very good (top 20%) or bad (bottom 20%) quality within the quantitative omics datasets on the basis of SNR values to reduce the impact of datasets with extreme quality values. A total of 60 batches of data were retained, including 9 batches of DNA (SNV/indel) data, 4 batches of DNA methylation profiles, 4 batches of miRNA profiles, 13 batches of RNA profiles, 18 batches of proteomics data and 12 batches of metabolomics data. In each vertical integration, we drew a random batch from the above dataset for each omics type, which means that the vertically integrated results were not affected by problems that exist in horizontal integration, for example, batch effects.

### Horizontal (within-omics) integration methods

A total of six methods were used in this study to horizontally integrate multiple batches of data, including Absolute, Ratio, ComBat, Harmony, RUVg and *z* score.

#### Absolute

The Absolute method refers to direct integration of data after preprocessing such as normalization, log_2_ transformation, missing value filtering and imputation.

#### Ratio

Ratio-based scaling refers to converting the quantitative profiles to relative-scale profiles within each batch on a feature-by-feature basis. To obtain ratio-based scaling data, the log_2_-transformed profiles for each feature of study samples (that is, D5, F7 and M8) were subtracted from the mean log_2_-transformed profiles of three replicates of the reference sample within the same batch (that is, D6).

#### ComBat

ComBat applies empirical Bayes shrinkage to adjust the mean and variance by pooling information across multiple genes to correct batch effects. It was implemented by using the ComBat function of the sva v3.38.0 package^106^ to adjust the known batches.

#### Harmony

Harmony uses an iterative clustering–correction procedure based on soft clustering to correct for sample differences. The algorithm was implemented by using the HarmonyMatrix function of the harmony v0.1.0 package^75^ with the parameter do_pca set to FALSE and other parameters as default.

#### RUVg

RUVg uses a subset of the data to estimate factors of unwanted variation. We used the least significant DEFs (25% of the total features) as ‘in silico empirical’ negative controls, which were considered not differentially expressed with the covariates of interest. Subsequently, we applied the RUVg function of the RUVSeq v1.24.0 package^76^ with default parameter settings.

#### *z* score

*z* score was performed by scaling each batch by feature before merging multiple batches to eliminate the batch effect.

### Vertical (cross-omics) integration methods

SNF^5^, iClusterBayes^77^, MOFA+^78^, MCIA^79^ and intNMF^80^ were used to integrate the multi-omics data. To reduce the impact of large differences in dimensionality across multi-omics datasets on the final results, for each omics type we selected the top 1,000 most variable features on the basis of the standard deviation. Subsequently, the data matrices were centered and scaled to a mean of 0 and a standard deviation of 1 feature by feature. In addition, because intNMF and MCIA are methods based on the principle of non-negative matrix decomposition, features containing negative values were added with their absolute of minimum values to ensure non-negativity.

The obtained sample labels were used to calculate ARI and the sample similarity matrix for the calculation of SNR. Because MOFA+ and MCIA do not provide clustering information, we applied PAM clustering to obtain the predicted sample labels. In addition to SNF, we used the affinityMatrix function in the SNFtools v2.3.1 package to process the results from iClusterBayes, MOFA+, MCIA and intNMF to derive the sample similarity matrices. The following vertical integration algorithms were used.

#### SNF

Similarity network fusion (SNF) constructs networks of samples for each available data type and then fuses these into one network that represents the full spectrum of underlying data. The SNFtool v2.3.1 package was used with the parameter K (number of neighbors) set to the square of the sample size after rounding, alpha (a hyperparameter) set to 0.5 and T (the number of iterations for the diffusion process) set to 10. The SNF-combined similarity matrix was directly used to calculate SNR.

#### iClusterBayes

iClusterBayes fits a Bayesian latent variable model on the basis of multi-omics data to identify a comprehensive cluster assignment, as well as latent variable features that contribute to clustering. This method was conducted with the iClusterBayes function of the iClusterPlus v1.26.0 package with the parameter K (number of eigen features) set to the number of sample groups minus one and other parameters as default. The latent variable was used to calculate the sample similarity matrix.

#### MOFA+

Multi-omics factor analysis v2 (MOFA+) reconstructs a low-dimensional representation of data using variational inference in terms of latent factors that capture the global sources of variability. The MOFA2 v1.1.21 package was used with the default parameters. The latent factors from the model were used to obtain the sample labels and similarity matrix.

#### MCIA

Multiple covariance analysis (MCIA) is an extension of covariance analysis (CIA) to multi-omics datasets. It was implemented by using the mcia function of the omicade4 v1.30.0 package. We obtained the sample labels and similarity matrix on the basis of the synthetic scores.

#### intNMF

intNMF is an extension of non-negative matrix factorization (NMF), which decomposes each omics dataset into the product of a factor matrix and an omics-specific weight matrix. This method was implemented using the intNMF v1.2.0 package with default parameters. The sample similarity matrix was obtained from the common basis matrix (*W*) across the multiple datasets.

### Identification and annotation of the multi-omics features associated with DNMs and DEFs

On the basis of the genomic coordinates of 2,187 DNMs^66^, we performed gene annotation using gencode.v40.annotation.gtf. The obtained gene list was applied to feature selection in other omics types. For methylation, we characterized the methylation level by taking the average *M* value in the promoter region (TSS200 or TSS1500) for these genes. miRNAs targeting these genes were obtained from the miRDB^102^ (prediction score of ≥80), miRTarBase^103^ (support type is ‘Functional MTI’) and TargetScan^104^ databases, and results appearing in all three databases were retained as miRNAs associated with these DNMs. RNA profiles were associated with gene names through Ensembl ID. Proteins were directly screened because they had been annotated to gene symbols. Finally, for metabolomics data, which are least closely associated with the genome from the central dogma, we obtained metabolites associated with the genes mentioned above from biological pathway information provided by the HMDB database^105^.

DEFs for D5 and D6 were identified in the same way as in the construction of reference datasets for DEFs, as detailed in the ‘Workflow for construction of reference datasets’ section.

For the features found in each omics type that differed between D5 and D6 using the two methods described above, we assessed intersections to obtain reliable multi-omics features affected by genomic-level differences between the identical twins (Fig. 6d). These features were further annotated with Ingenuity Pathway Analysis software to obtain candidate biological pathways associated with these features. Ultimately, pathways associated with more than one feature and with Fisher’s exact test *P* < 0.05 were retained (Fig. 6e).

### Similarity between D5 and D6

The SNF method was used to integrate data from different multi-omics combinations to explore the genomic inheritance patterns of the Quartet identical twins during data integration. During integration, we randomly selected a batch from each omics type with moderate quality (SNR in the range of 20% to 80%) and then calculated the inter-sample similarity matrix *W* using the SNFtool v2.3.1 package. Specifically, for single-omics datasets (that is, DNA or metabolites), we treated two random batches of moderate-quality data from the same omics type as different sources, also using SNF for integration and to obtain the *W* matrix. As D5 and D6 each had three technical replicates, there were nine similarity results in the *W* matrix. We used their mean values as the similarity between D5 and D6 obtained from one integration. A total of 50 iterations were performed to ensure robustness.

### Statistical analysis

All statistical analyses were performed using R statistical packages (version 4.0.5) (https://www.r-project.org). Pearson’s correlation coefficients were calculated using the Hmisc v4.6.0 package (https://CRAN.R-project.org/package=Hmisc). Differential expression analyses were implemented using the ChAMP v2.20.1 package for EPIC methylation data^89^ and using the rstatix v0.7.0 package for other omics data (https://github.com/kassambara/rstatix). PCA was conducted with univariance scaling using the prcomp function. PAM clustering was implemented using the cluster v2.1.3 package (https://CRAN.R-project.org/package=cluster). Data visualization was implemented using the R packages ggplot2 v3.3.6 (https://ggplot2.tidyverse.org/), ggsci v2.9 (https://github.com/nanxstats/ggsci), ggpubr v0.4.0 (https://github.com/kassambara/ggpubr/), ComplexHeatmap v2.6.2 (ref. ^107^) and networkD3 v0.4 (https://christophergandrud.github.io/networkD3/).

### Materials availability

The Quartet multi-omics reference materials generated in this study can be accessed from the Quartet Data Portal (https://chinese-quartet.org/) under the Administrative Regulations of the People’s Republic of China on Human Genetic Resources.

### Reporting summary

Further information on research design is available in the Nature Portfolio Reporting Summary linked to this article.

## Online content

Any methods, additional references, Nature Portfolio reporting summaries, source data, extended data, supplementary information, acknowledgements, peer review information; details of author contributions and competing interests; and statements of data and code availability are available at 10.1038/s41587-023-01934-1.

## Supplementary information

Supplementary information Supplementary Figs. 1–3.

Reporting Summary

Supplementary Tables .Supplementary Tables 1–4.

Supplementary Data Supplementary Data 1–5.

## Figures and Tables

**Extended Data Table 1:**
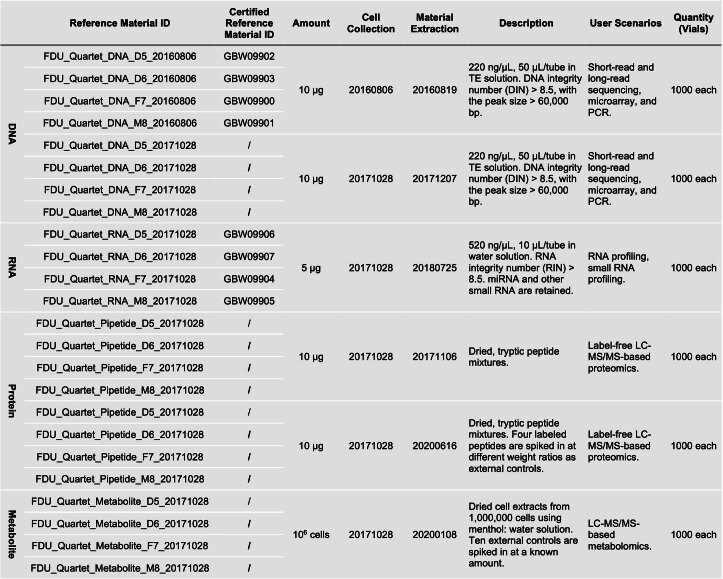
Summary of Quartet multi-omics reference materials

The Quartet multi-omics reference materials are from a monozygotic twin family, including father (F7), mother (M8), and monozygotic twin daughters (D5 and D6).

**Extended Data Table 2:**
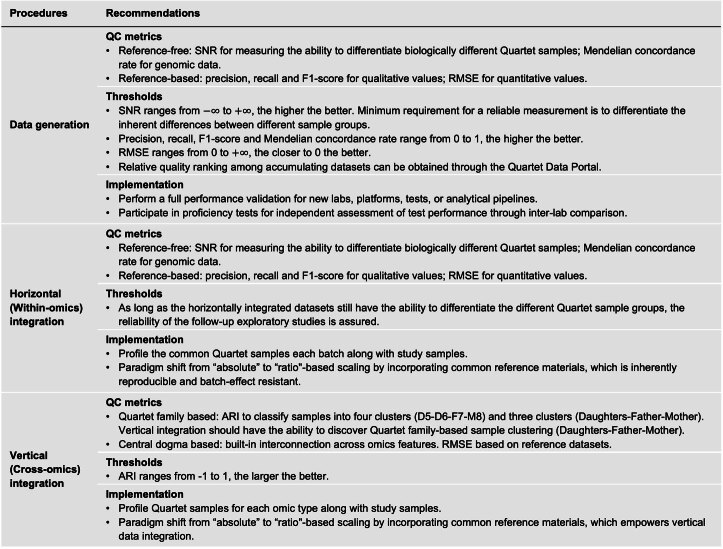
Best practices for QC using Quartet reference materials

QC metrics, thresholds, and implementation for multi-omics data generation, horizontal (within-omics) integration, and vertical (cross-omics) integration. SNR: Signal-to-Noise Ratio; RMSE: Root Mean Square Error; ARI: Adjusted Rand Index.

## Data Availability

All raw data, processed data and reference datasets can be accessed from the Quartet Data Portal (https://chinese-quartet.org/) under the Administrative Regulations of the People’s Republic of China on Human Genetic Resources. They can also be accessed from the Genome Sequence Archive (GSA), Genome Variation Map (GVM) and Open Archive for Miscellaneous Data (OMIX) of the National Genomics Data Center of China with BioProject ID PRJCA012423 (ref. ^108^).

## Code availability

The source code for data analysis and figure generation has been deposited on Zenodo^109^. The source code for quality assessment based on Quartet multi-omics reference materials and reference datasets is available on GitHub^110^.

## References

[1] Hasin, Y., Seldin, M. & Lusis, A. Multi-omics approaches to disease. *Genome Biol.* **18**, 83 (2017). 28476144 10.1186/s13059-017-1215-1 PMC5418815

[2] Karczewski, K. J. & Snyder, M. P. Integrative omics for health and disease. *Nat. Rev. Genet.* **19**, 299–310 (2018). 29479082 10.1038/nrg.2018.4 PMC5990367

[3] Shilo, S., Rossman, H. & Segal, E. Axes of a revolution: challenges and promises of big data in healthcare. *Nat. Med.* **26**, 29–38 (2020). 31932803 10.1038/s41591-019-0727-5

[4] Ideker, T., Galitski, T. & Hood, L. A new approach to decoding life: systems biology. *Annu. Rev. Genom. Hum. Genet.* **2**, 343–372 (2001). 10.1146/annurev.genom.2.1.343 11701654

[5] Wang, B. et al. Similarity network fusion for aggregating data types on a genomic scale. *Nat. Methods* **11**, 333–337 (2014). 24464287 10.1038/nmeth.2810

[6] Yan, J., Risacher, S. L., Shen, L. & Saykin, A. J. Network approaches to systems biology analysis of complex disease: integrative methods for multi-omics data. *Brief. Bioinformatics* **19**, 1370–1381 (2018). 28679163 10.1093/bib/bbx066 PMC6454489

[7] Hawe, J. S., Theis, F. J. & Heinig, M. Inferring interaction networks from multi-omics data. *Front. Genet.* **10**, 535 (2019). 31249591 10.3389/fgene.2019.00535 PMC6582773

[8] Yurkovich, J. T., Tian, Q., Price, N. D. & Hood, L. A systems approach to clinical oncology uses deep phenotyping to deliver personalized care. *Nat. Rev. Clin. Oncol.* **17**, 183–194 (2020). 31619755 10.1038/s41571-019-0273-6

[9] Chang, K. et al. The Cancer Genome Atlas Pan-Cancer analysis project. *Nat. Genet.* **45**, 1113–1120 (2013). 24071849 10.1038/ng.2764 PMC3919969

[10] Bycroft, C. et al. The UK Biobank resource with deep phenotyping and genomic data. *Nature* **562**, 203–209 (2018). 30305743 10.1038/s41586-018-0579-z PMC6786975

[11] Campbell, P. J. et al. Pan-cancer analysis of whole genomes. *Nature* **578**, 82–93 (2020). 32025007 10.1038/s41586-020-1969-6 PMC7025898

[12] Denny, J. C. & Collins, F. S. Precision medicine in 2030—seven ways to transform healthcare. *Cell* **184**, 1415–1419 (2021). 33740447 10.1016/j.cell.2021.01.015 PMC9616629

[13] Jin, L. Welcome to the phenomics. *J. Phenomics* **1**, 1–2 (2021). 10.1007/s43657-020-00009-4 PMC9584128 36939790

[14] Veturi, Y. et al. A unified framework identifies new links between plasma lipids and diseases from electronic medical records across large-scale cohorts. *Nat. Genet.* **53**, 972–981 (2021). 34140684 10.1038/s41588-021-00879-y PMC8555954

[15] Tarazona, S., Arzalluz-Luque, A. & Conesa, A. Undisclosed, unmet and neglected challenges in multi-omics studies. *Nat. Comput. Sci.* **1**, 395–402 (2021). 38217236 10.1038/s43588-021-00086-z

[16] Burk, R. D. et al. Integrated genomic and molecular characterization of cervical cancer. *Nature* **543**, 378–384 (2017). 28112728 10.1038/nature21386 PMC5354998

[17] Jiang, Y. Z. et al. Genomic and transcriptomic landscape of triple-negative breast cancers: subtypes and treatment strategies. *Cancer Cell* **35**, 428–440 (2019). 30853353 10.1016/j.ccell.2019.02.001

[18] Zimmer, A. et al. The geometry of clinical labs and wellness states from deeply phenotyped humans. *Nat. Commun.* **12**, 3578 (2021). 34117230 10.1038/s41467-021-23849-8 PMC8196202

[19] Menyhárt, O. & Győrffy, B. Multi-omics approaches in cancer research with applications in tumor subtyping, prognosis, and diagnosis. *Comput. Struct. Biotechnol. J.* **19**, 949–960 (2021). 33613862 10.1016/j.csbj.2021.01.009 PMC7868685

[20] Zhou, W. et al. Longitudinal multi-omics of host–microbe dynamics in prediabetes. *Nature* **569**, 663–671 (2019). 31142858 10.1038/s41586-019-1236-x PMC6666404

[21] Contrepois, K. et al. Molecular choreography of acute exercise. *Cell* **181**, 1112–1130 (2020). 32470399 10.1016/j.cell.2020.04.043 PMC7299174

[22] Li, Y. et al. Using composite phenotypes to reveal hidden physiological heterogeneity in high-altitude acclimatization in a Chinese Han longitudinal cohort. *Phenomics* **1**, 3–14 (2021). 36939745 10.1007/s43657-020-00005-8 PMC9584130

[23] Lehmann, B. D. et al. Multi-omics analysis identifies therapeutic vulnerabilities in triple-negative breast cancer subtypes. *Nat. Commun.* **12**, 6276 (2021). 34725325 10.1038/s41467-021-26502-6 PMC8560912

[24] Schulte-Sasse, R., Budach, S., Hnisz, D. & Marsico, A. Integration of multiomics data with graph convolutional networks to identify new cancer genes and their associated molecular mechanisms. *Nat. Mach. Intell.* **3**, 513–526 (2021).

[25] Silverbush, D. et al. Simultaneous integration of multi-omics data improves the identification of cancer driver modules. *Cell Syst.* **8**, 456–466 (2019). 31103572 10.1016/j.cels.2019.04.005

[26] Price, N. D. et al. A wellness study of 108 individuals using personal, dense, dynamic data clouds. *Nat. Biotechnol.* **35**, 747–756 (2017). 28714965 10.1038/nbt.3870 PMC5568837

[27] Tebani, A. et al. Integration of molecular profiles in a longitudinal wellness profiling cohort. *Nat. Commun.* **11**, 4487 (2020). 32900998 10.1038/s41467-020-18148-7 PMC7479148

[28] Wilmanski, T. et al. Blood metabolome predicts gut microbiome α-diversity in humans. *Nat. Biotechnol.* **37**, 1217–1228 (2019). 31477923 10.1038/s41587-019-0233-9

[29] Dodig-Crnković, T. et al. Facets of individual-specific health signatures determined from longitudinal plasma proteome profiling. *EBioMedicine* **57**, 102854 (2020). 32629387 10.1016/j.ebiom.2020.102854 PMC7334812

[30] Leiserson, M. D. M. et al. Pan-cancer network analysis identifies combinations of rare somatic mutations across pathways and protein complexes. *Nat. Genet.* **47**, 106–114 (2015). 25501392 10.1038/ng.3168 PMC4444046

[31] Schüssler-Fiorenza Rose, S. M. et al. A longitudinal big data approach for precision health. *Nat. Med.* **25**, 792–804 (2019). 31068711 10.1038/s41591-019-0414-6 PMC6713274

[32] Tarazona, S. et al. Harmonization of quality metrics and power calculation in multi-omic studies. *Nat. Commun.* **11**, 3092 (2020). 32555183 10.1038/s41467-020-16937-8 PMC7303201

[33] Palsson, B. & Zengler, K. The challenges of integrating multi-omic data sets. *Nat. Chem. Biol.* **6**, 787–789 (2010). 20976870 10.1038/nchembio.462

[34] Argelaguet, R., Cuomo, A. S. E., Stegle, O. & Marioni, J. C. Computational principles and challenges in single-cell data integration. *Nat. Biotechnol.* **39**, 1202–1215 (2021). 33941931 10.1038/s41587-021-00895-7

[35] Leek, J. T. et al. Tackling the widespread and critical impact of batch effects in high-throughput data. *Nat. Rev. Genet.* **11**, 733–739 (2010). 20838408 10.1038/nrg2825 PMC3880143

[36] Goh, W. W. B., Wang, W. & Wong, L. Why batch effects matter in omics data, and how to avoid them. *Trends Biotechnol.* **35**, 498–507 (2017). 28351613 10.1016/j.tibtech.2017.02.012

[37] Zhou, L., Chi-Hau Sue, A. & Bin Goh, W. W. Examining the practical limits of batch effect-correction algorithms: when should you care about batch effects? *J. Genet. Genomics* **46**, 433–443 (2019). 31611172 10.1016/j.jgg.2019.08.002

[38] Luecken, M. D. et al. Benchmarking atlas-level data integration in single-cell genomics. *Nat. Methods* **19**, 41–50 (2022). 34949812 10.1038/s41592-021-01336-8 PMC8748196

[39] Tran, H. T. N. et al. A benchmark of batch-effect correction methods for single-cell RNA sequencing data. *Genome Biol.* **21**, 12 (2020). 31948481 10.1186/s13059-019-1850-9 PMC6964114

[40] Misra, B. B., Langefeld, C., Olivier, M. & Cox, L. A. Integrated omics: tools, advances and future approaches. *J. Mol. Endocrinol.* **62**, R21–R45 (2019). 10.1530/JME-18-0055 30006342

[41] Krassowski, M., Das, V., Sahu, S. K. & Misra, B. B. State of the field in multi-omics research: from computational needs to data mining and sharing. *Front. Genet.* **11**, 610798 (2020). 33362867 10.3389/fgene.2020.610798 PMC7758509

[42] Cantini, L. et al. Benchmarking joint multi-omics dimensionality reduction approaches for the study of cancer. *Nat. Commun.* **12**, 124 (2021). 33402734 10.1038/s41467-020-20430-7 PMC7785750

[43] Rappoport, N. & Shamir, R. Multi-omic and multi-view clustering algorithms: review and cancer benchmark. *Nucleic Acids Res.* **47**, 1044 (2019). 30496480 10.1093/nar/gky1226 PMC6344869

[44] Choobdar, S. et al. Assessment of network module identification across complex diseases. *Nat. Methods* **16**, 843–852 (2019). 31471613 10.1038/s41592-019-0509-5 PMC6719725

[45] Sené, M., Gilmore, I. & Janssen, J. T. Metrology is key to reproducing results. *Nature* **547**, 397–399 (2017). 28748943 10.1038/547397a

[46] Hardwick, S. A., Deveson, I. W. & Mercer, T. R. Reference standards for next-generation sequencing. *Nat. Rev. Genet.* **18**, 473–484 (2017). 28626224 10.1038/nrg.2017.44

[47] Salit, M. & Woodcock, J. MAQC and the era of genomic medicine. *Nat. Biotechnol.* **39**, 1066–1067 (2021). 34504343 10.1038/s41587-021-01050-y

[48] Choquette, S. J., Duewer, D. L. & Sharpless, K. E. NIST reference materials: utility and future. *Annu. Rev. Anal. Chem.* **13**, 453–474 (2020). 10.1146/annurev-anchem-061318-115314 32176531

[49] Zook, J. M. et al. An open resource for accurately benchmarking small variant and reference calls. *Nat. Biotechnol.* **37**, 561–566 (2019). 30936564 10.1038/s41587-019-0074-6 PMC6500473

[50] Zook, J. M. et al. A robust benchmark for detection of germline large deletions and insertions. *Nat. Biotechnol.* **38**, 1347–1355 (2020). 32541955 10.1038/s41587-020-0538-8 PMC8454654

[51] Jones, W. et al. A verified genomic reference sample for assessing performance of cancer panels detecting small variants of low allele frequency. *Genome Biol.* **22**, 111 (2021). 33863366 10.1186/s13059-021-02316-z PMC8051128

[52] Deveson, I. W. et al. Evaluating the analytical validity of circulating tumor DNA sequencing assays for precision oncology. *Nat. Biotechnol.* **39**, 1115–1128 (2021). 33846644 10.1038/s41587-021-00857-z PMC8434938

[53] Fang, L. T. et al. Establishing community reference samples, data and call sets for benchmarking cancer mutation detection using whole-genome sequencing. *Nat. Biotechnol.* **39**, 1151–1160 (2021). 34504347 10.1038/s41587-021-00993-6 PMC8532138

[54] Su, Z. et al. A comprehensive assessment of RNA-seq accuracy, reproducibility and information content by the Sequencing Quality Control Consortium. *Nat. Biotechnol.* **32**, 903–914 (2014). 25150838 10.1038/nbt.2957 PMC4321899

[55] Shi, L. et al. The MicroArray Quality Control (MAQC) project shows inter- and intraplatform reproducibility of gene expression measurements. *Nat. Biotechnol.* **24**, 1151–1161 (2006). 16964229 10.1038/nbt1239 PMC3272078

[56] Friedman, D. B. et al. The ABRF Proteomics Research Group studies: educational exercises for qualitative and quantitative proteomic analyses. *Proteomics* **11**, 1371–1381 (2011). 21394914 10.1002/pmic.201000736

[57] Ulmer, C. Z. et al. LipidQC: method validation tool for visual comparison to SRM 1950 using NIST interlaboratory comparison exercise lipid consensus mean estimate values. *Anal. Chem.* **89**, 13069–13073 (2017). 29148710 10.1021/acs.analchem.7b04042 PMC11493136

[58] Krusche, P. et al. Best practices for benchmarking germline small-variant calls in human genomes. *Nat. Biotechnol.* **37**, 555–560 (2019). 30858580 10.1038/s41587-019-0054-x PMC6699627

[59] Matthijs, G. et al. Guidelines for diagnostic next-generation sequencing. *Eur. J. Hum. Genet.* **24**, 1515 (2016). 27628564 10.1038/ejhg.2016.63 PMC5027692

[60] Gargis, A. S. et al. Assuring the quality of next-generation sequencing in clinical laboratory practice. *Nat. Biotechnol.* **30**, 1033–1036 (2012). 23138292 10.1038/nbt.2403 PMC3827024

[61] Broadhurst, D. et al. Guidelines and considerations for the use of system suitability and quality control samples in mass spectrometry assays applied in untargeted clinical metabolomic studies. *Metabolomics* **14**, 72 (2018). 29805336 10.1007/s11306-018-1367-3 PMC5960010

[62] Collins, B. C. et al. Multi-laboratory assessment of reproducibility, qualitative and quantitative performance of SWATH-mass spectrometry. *Nat. Commun.* **8**, 291 (2017). 28827567 10.1038/s41467-017-00249-5 PMC5566333

[63] Beger, R. D. et al. Towards quality assurance and quality control in untargeted metabolomics studies. *Metabolomics* **15**, 4 (2019). 30830465 10.1007/s11306-018-1460-7 PMC6443086

[64] Wang, X. et al. QC metrics from CPTAC raw LC–MS/MS data interpreted through multivariate statistics. *Anal. Chem.* **86**, 2497–2509 (2014). 24494671 10.1021/ac4034455 PMC3982976

[65] Chen, X. D., Jiang, Y. F., Xu, P. & Jin, L. Construction and utilization of human genetic resources in large population cohorts. *Yi Chuan* **43**, 980–987 (2021). 34702710 10.16288/j.yczz.21-195

[66] Ren, L. et al. Quartet DNA reference materials and datasets for comprehensively evaluating germline variants calling performance. Preprint at *bioRxiv*10.1101/2022.09.28.509844 (2022). 10.1186/s13059-023-03109-2 PMC10680274 38012772

[67] Yu, Y. et al. Quartet RNA reference materials improve the quality of transcriptomic data through ratio-based profiling. *Nat. Biotechnol.*10.1038/s41587-023-01867-9 (2023). 10.1038/s41587-023-01867-9 PMC11251996 37679545

[68] Tian, S. et al. Quartet protein reference materials and datasets for multi-platform assessment of label-free proteomics. *Genome Biol.* (in the press). 10.1186/s13059-023-03048-y PMC10483797 37674236

[69] Zhang, N. et al. Quartet metabolite reference materials for assessing inter-laboratory reliability and data integration of metabolomic profiling. Preprint at *bioRxiv*10.1101/2022.11.01.514762 (2022).

[70] Jia, P. et al. Haplotype-resolved assemblies and variant benchmark of a Chinese Quartet. Preprint at *bioRxiv*10.1101/2022.09.08.504083 (2022). 10.1186/s13059-023-03116-3 PMC10694985 38049885

[71] Yu, Y. et al. Correcting batch effects in large-scale multiomic studies using a reference-material-based ratio method. *Genome Biol.* (in the press). 10.1186/s13059-023-03047-z PMC10483871 37674217

[72] Yang, J. et al. The Quartet Data Portal: integration of community-wide resources for multiomics quality control. *Genome Biol.* (in the press). 10.1186/s13059-023-03091-9 PMC10601216 37884999

[73] Heumos, L. et al. Best practices for single-cell analysis across modalities. *Nat. Rev. Genet*. **24**, 550–572 (2023). 10.1038/s41576-023-00586-w PMC10066026 37002403

[74] Zhang, Y., Parmigiani, G. & Johnson, W. E. ComBat-seq: batch effect adjustment for RNA-seq count data. *NAR Genom. Bioinform.* **2**, lqaa078 (2020). 33015620 10.1093/nargab/lqaa078 PMC7518324

[75] Korsunsky, I. et al. Fast, sensitive and accurate integration of single-cell data with Harmony. *Nat. Methods* **16**, 1289–1296 (2019). 31740819 10.1038/s41592-019-0619-0 PMC6884693

[76] Risso, D., Ngai, J., Speed, T. P. & Dudoit, S. Normalization of RNA-seq data using factor analysis of control genes or samples. *Nat. Biotechnol.* **32**, 896–902 (2014). 25150836 10.1038/nbt.2931 PMC4404308

[77] Mo, Q. et al. A fully Bayesian latent variable model for integrative clustering analysis of multi-type omics data. *Biostatistics* **19**, 71–86 (2017). 10.1093/biostatistics/kxx017 PMC6455926 28541380

[78] Argelaguet, R. et al. MOFA+: a statistical framework for comprehensive integration of multi-modal single-cell data. *Genome Biol.* **21**, 111 (2020). 32393329 10.1186/s13059-020-02015-1 PMC7212577

[79] Meng, C., Kuster, B., Culhane, A. C. & Gholami, A. M. A multivariate approach to the integration of multi-omics datasets. *BMC Bioinformatics* **15**, 162 (2014). 24884486 10.1186/1471-2105-15-162 PMC4053266

[80] Chalise, P. & Fridley, B. L. Integrative clustering of multi-level ‘omic data based on non-negative matrix factorization algorithm. *PLoS ONE* **12**, e0176278 (2017). 28459819 10.1371/journal.pone.0176278 PMC5411077

[81] Hubert, L. & Arabie, P. Comparing partitions. *J. Classif.* **2**, 193–218 (1985).

[82] Schubert, E. & Rousseeuw, P. J. Fast and eager *k*-medoids clustering: *O* (*k*) runtime improvement of the PAM, CLARA, and CLARANS algorithms. *Inf. Syst.* **101**, 101804 (2021).

[83] Baker, M. 1,500 scientists lift the lid on reproducibility. *Nature* **533**, 452–454 (2016). 27225100 10.1038/533452a

[84] Giraldez, M. D. et al. Comprehensive multi-center assessment of small RNA-seq methods for quantitative miRNA profiling. *Nat. Biotechnol.* **36**, 746–757 (2018). 30010675 10.1038/nbt.4183 PMC6078798

[85] Luo, J. et al. A comparison of batch effect removal methods for enhancement of prediction performance using MAQC-II microarray gene expression data. *Pharmacogenomics J.* **10**, 278–291 (2010). 20676067 10.1038/tpj.2010.57 PMC2920074

[86] Shi, L. et al. Microarray scanner calibration curves: characteristics and implications. *BMC Bioinformatics* **6**, S11 (2005). 16026596 10.1186/1471-2105-6-S2-S11 PMC1637029

[87] Chen, J. J., Hsueh, H.-M., Delongchamp, R. R., Lin, C.-J. & Tsai, C.-A. Reproducibility of microarray data: a further analysis of microarray quality control (MAQC) data. *BMC Bioinformatics* **8**, 412 (2007). 17961233 10.1186/1471-2105-8-412 PMC2204045

[88] Wheeler, H. E. & Dolan, M.E. Lymphoblastoid cell lines in pharmacogenomic discovery and clinical translation. *Pharmacogenomics* **13**, 55–70 (2012). 22176622 10.2217/pgs.11.121 PMC3292907

[89] Tian, Y. et al. ChAMP: updated methylation analysis pipeline for Illumina BeadChips. *Bioinformatics* **33**, 3982–3984 (2017). 28961746 10.1093/bioinformatics/btx513 PMC5860089

[90] Aryee, M. J. et al. Minfi: a flexible and comprehensive Bioconductor package for the analysis of Infinium DNA methylation microarrays. *Bioinformatics* **30**, 1363–1369 (2014). 24478339 10.1093/bioinformatics/btu049 PMC4016708

[91] Fortin, J.-P., Triche, T. J. Jr & Hansen, K. D. Preprocessing, normalization and integration of the Illumina HumanMethylationEPIC array with minfi. *Bioinformatics* **33**, 558–560 (2017). 28035024 10.1093/bioinformatics/btw691 PMC5408810

[92] Triche, T. J. Jr, Weisenberger, D. J., Van Den Berg, D., Laird, P. W. & Siegmund, K. D. Low-level processing of Illumina Infinium DNA methylation beadarrays. *Nucleic Acids Res.* **41**, e90 (2013). 23476028 10.1093/nar/gkt090 PMC3627582

[93] Kim, D., Paggi, J. M., Park, C., Bennett, C. & Salzberg, S. L. Graph-based genome alignment and genotyping with HISAT2 and HISAT-genotype. *Nat. Biotechnol.* **37**, 907–915 (2019). 31375807 10.1038/s41587-019-0201-4 PMC7605509

[94] Li, H. A statistical framework for SNP calling, mutation discovery, association mapping and population genetical parameter estimation from sequencing data. *Bioinformatics* **27**, 2987–2993 (2011). 21903627 10.1093/bioinformatics/btr509 PMC3198575

[95] Pertea, M. et al. StringTie enables improved reconstruction of a transcriptome from RNA-seq reads. *Nat. Biotechnol.* **33**, 290–295 (2015). 25690850 10.1038/nbt.3122 PMC4643835

[96] Rozowsky, J. et al. exceRpt: a comprehensive analytic platform for extracellular RNA profiling. *Cell Syst.* **8**, 352–357 (2019). 30956140 10.1016/j.cels.2019.03.004 PMC7079576

[97] Feng, J. et al. Firmiana: towards a one-stop proteomic cloud platform for data processing and analysis. *Nat. Biotechnol.* **35**, 409–412 (2017). 28486446 10.1038/nbt.3825

[98] Josse, J. & Husson, F. missMDA: a package for handling missing values in multivariate data analysis. *J. Stat. Softw.* **70**, 1–31 (2016).

[99] Bates, D., Mächler, M., Bolker, B. & Walker, S. Fitting linear mixed-effects models using lme4. *J. Stat. Softw.* **67**, 1–48 (2015).

[100] Li, Y., Ge, X., Peng, F., Li, W. & Li, J. J. Exaggerated false positives by popular differential expression methods when analyzing human population samples. *Genome Biol.* **23**, 79 (2022). 35292087 10.1186/s13059-022-02648-4 PMC8922736

[101] Guo, L. et al. Rat toxicogenomic study reveals analytical consistency across microarray platforms. *Nat. Biotechnol.* **24**, 1162–1169 (2006). 17061323 10.1038/nbt1238

[102] Wong, N. & Wang, X. miRDB: an online resource for microRNA target prediction and functional annotations. *Nucleic Acids Res.* **43**, D146–D152 (2015). 25378301 10.1093/nar/gku1104 PMC4383922

[103] Huang, H.-Y. et al. miRTarBase 2020: updates to the experimentally validated microRNA–target interaction database. *Nucleic Acids Res.* **48**, D148–D154 (2020). 31647101 10.1093/nar/gkz896 PMC7145596

[104] McGeary, S. E. et al. The biochemical basis of microRNA targeting efficacy. *Science* **366**, eaav1741 (2019). 31806698 10.1126/science.aav1741 PMC7051167

[105] Wishart, D. S. et al. HMDB 5.0: the human metabolome database for 2022. *Nucleic Acids Res.* **50**, D622–D631 (2022). 34986597 10.1093/nar/gkab1062 PMC8728138

[106] Leek, J. T., Johnson, W. E., Parker, H. S., Jaffe, A. E. & Storey, J. D. The sva package for removing batch effects and other unwanted variation in high-throughput experiments. *Bioinformatics* **28**, 882–883 (2012). 22257669 10.1093/bioinformatics/bts034 PMC3307112

[107] Gu, Z., Eils, R. & Schlesner, M. Complex heatmaps reveal patterns and correlations in multidimensional genomic data. *Bioinformatics* **32**, 2847–2849 (2016). 27207943 10.1093/bioinformatics/btw313

[108] Quartet Project Team. Quartet Project for quality control and data integration of multi-omics profiling. *National Genomics Data Center* https://ngdc.cncb.ac.cn/bioproject/browse/PRJCA012423 (2023).

[109] Liu, Y. Multi-omics data integration using ratio-based quantitative profiling with Quartet reference materials. *Zenodo*10.5281/zenodo.8185817 (2023). 10.1038/s41587-023-01934-1 PMC11252085 37679543

[110] Quartet Project Team. Chinese Quartet. *GitHub* https://github.com/chinese-quartet (2023).

