# Supplementary material for: Multi-omics data integration using ratio-based quantitative profiling with Quartet reference materials
Source: Nat Biotechnol. 2023 Sep 7;42(7):1133–49. doi: 10.1038/s41587-023-01934-1 (PMC11252085; doi:10.1038/s41587-023-01934-1)
Supplement: Supplementary file 1 — Supplementary Figs. 1–3. [file 41587_2023_1934_MOESM1_ESM.pdf]

nature biotechnology

Article https://doi.org/10.1038/s41587-023-01934-1

Multi-omics data integration using ratio-based quantitative profiling with Quartet reference materials

In the format provided by the authors and unedited

Multi-omics data integration using ratio-based quantitative profiling with Quartet reference materials

Supplementary information

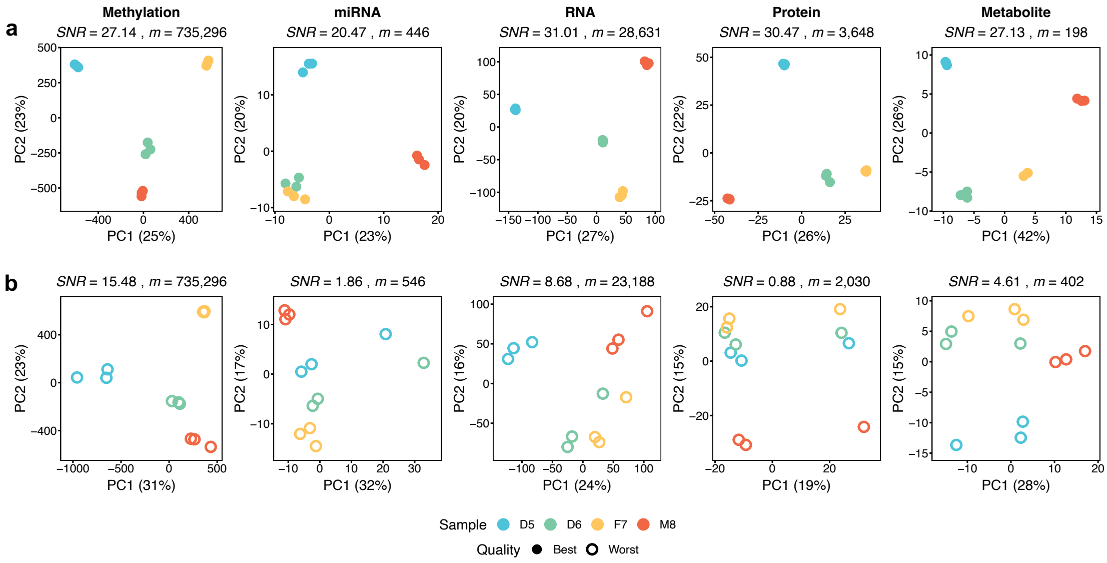

Supplementary Fig. 1 | Quartet multi-sample based intra-batch Signal-to-Noise Ratio (SNR) for performance evaluation of each omics profiling.

Intra-batch performance evaluation using SNR. Two batches of typically good (**a**) and bad (**b**) quality datasets of methylomics, transcriptomics, proteomics, and metabolomics were visualized by PCA plots. *m* is the number of features of the matrix used to calculate the SNR for the batch.

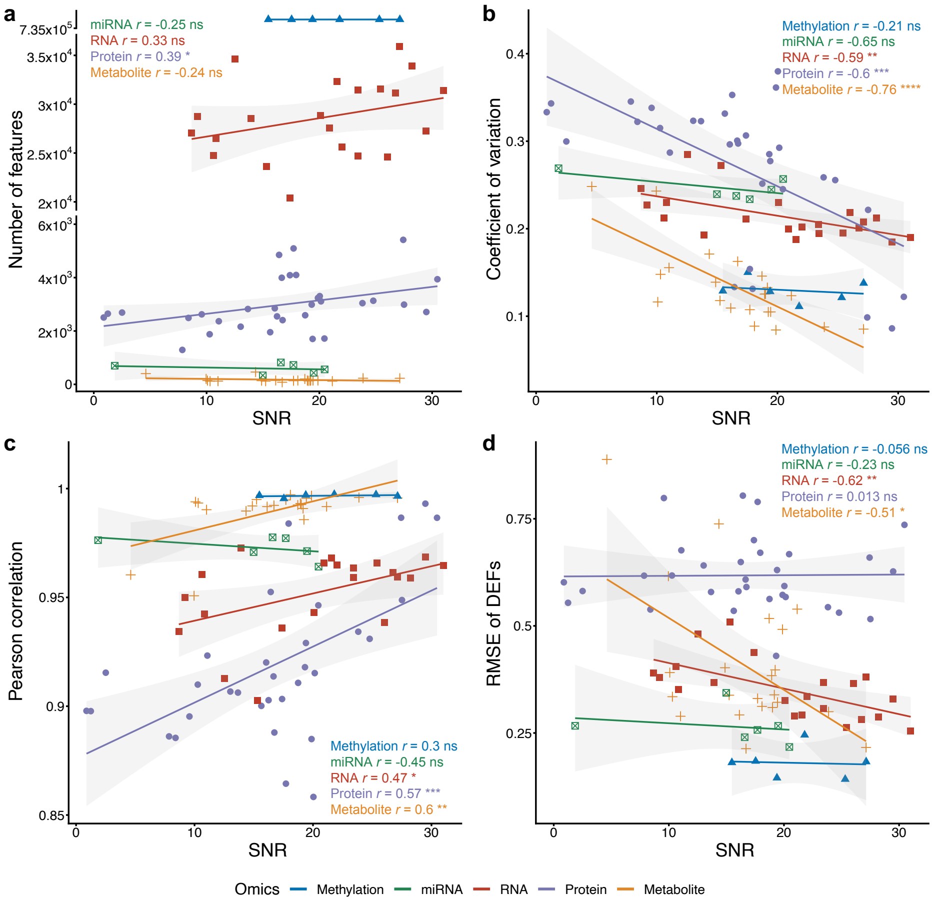

Supplementary Fig. 2 | Scatter plots between SNR and the number of features, CV, Pearson correlation, and RMSE.

Data points represent one batch and solid lines indicate fitted lines obtained from linear regression. The shading indicates the 95% confidence interval around the smoothing. Red: Methylation; Blue: miRNA; Yellow: RNA; Green: Protein; Purple: Metabolite. The annotated correlations were Pearson correlation coefficients. ns, $p \geq 0.05$ refers to not significant, * $p<0.05$, ** $p<0.01$, *** $p<0.001$, **** $p<0.0001$. Specific *p* values are listed in **Supplementary Data 5**. **a**, Scatter plots between SNR and the number of features. **b**, Scatter plots between SNR and Coefficient of Variation (CV). The CV for each batch is the mean value of the CVs between technical replicates on all features for the four sample groups. **c**, Scatter plots between SNR and Pearson correlation coefficient, which is the mean of the results of the two-by-two calculations of the three technical replicates for each sample group. **d**, Scatter plots between SNR and RMSE of DEFs, which is the mean of RMSEs of all features within one batch.

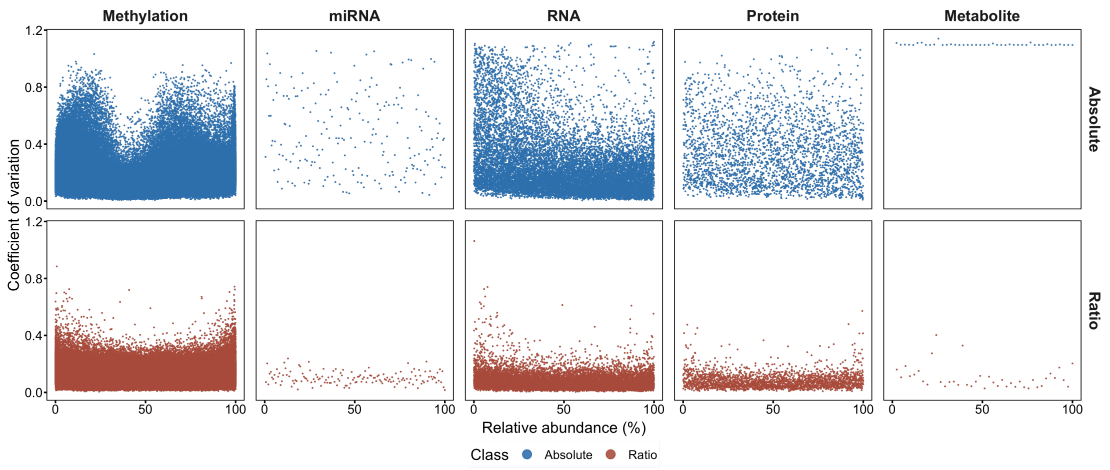

Supplementary Fig. 3 | Scatter plots between inter-batch technical replicates of D5 samples and feature abundance.

Data points represent the coefficient of variation (CV) of six technical replicates of D5 samples from two batches, measured at both the absolute level (Blue) and the ratio level (Red). The selected batches were of good quality and from different labs, which is consistent with **Fig. 3a**. The x-axis represents the relative abundance of the features, which are transformed into percentages. The closer the score is to 100%, the higher the abundance of that feature among all the features.
