# Supplementary material for: Multi-omics data integration using ratio-based quantitative profiling with Quartet reference materials
Source: Nat Biotechnol. 2023 Sep 7;42(7):1133–49. doi: 10.1038/s41587-023-01934-1 (PMC11252085; doi:10.1038/s41587-023-01934-1)
Supplement: Supplementary file 2 — Reporting Summary [file 41587_2023_1934_MOESM2_ESM.pdf]

nature portfolio

Corresponding author(s): Yuanting Zheng, Chen Ding, Jinming Li, Xiang Fang, Leming Shi

Last updated by author(s): Aug 30, 2023

Reporting Summary

Nature Portfolio wishes to improve the reproducibility of the work that we publish. This form provides structure for consistency and transparency in reporting. For further information on Nature Portfolio policies, see our Editorial Policies and the Editorial Policy Checklist.

Statistics

For all statistical analyses, confirm that the following items are present in the figure legend, table legend, main text, or Methods section.

n/a	Confirmed	
☐	☒	The exact sample size (n) for each experimental group/condition, given as a discrete number and unit of measurement
☐	☒	A statement on whether measurements were taken from distinct samples or whether the same sample was measured repeatedly
☐	☒	The statistical test(s) used AND whether they are one- or two-sided<br>*Only common tests should be described solely by name; describe more complex techniques in the Methods section.*
☒	☐	A description of all covariates tested
☐	☒	A description of any assumptions or corrections, such as tests of normality and adjustment for multiple comparisons
☐	☒	A full description of the statistical parameters including central tendency (e.g. means) or other basic estimates (e.g. regression coefficient) AND variation (e.g. standard deviation) or associated estimates of uncertainty (e.g. confidence intervals)
☐	☒	For null hypothesis testing, the test statistic (e.g. F, t, r) with confidence intervals, effect sizes, degrees of freedom and P value noted<br>*Give P values as exact values whenever suitable.*
☒	☐	For Bayesian analysis, information on the choice of priors and Markov chain Monte Carlo settings
☒	☐	For hierarchical and complex designs, identification of the appropriate level for tests and full reporting of outcomes
☐	☒	Estimates of effect sizes (e.g. Cohen's d, Pearson's r), indicating how they were calculated

Our web collection on statistics for biologists contains articles on many of the points above.

Software and code

Policy information about availability of computer code

Data collection	The details of methods for the multi-omics data collection were described in the Methods section. The source codes are available at Github (https://github.com/chinese-quartet/).
Data analysis	Software: R v4.0.5. R codes for data analysis and figure generation are deposited in Zenodo (https://doi.org/10.5281/zenodo.8176532).

For manuscripts utilizing custom algorithms or software that are central to the research but not yet described in published literature, software must be made available to editors and reviewers. We strongly encourage code deposition in a community repository (e.g. GitHub). See the Nature Portfolio guidelines for submitting code & software for further information.

Data

Policy information about availability of data

All manuscripts must include a data availability statement. This statement should provide the following information, where applicable:

- Accession codes, unique identifiers, or web links for publicly available datasets
- A description of any restrictions on data availability
- For clinical datasets or third party data, please ensure that the statement adheres to our policy

All the raw data, processed data, and reference datasets can be accessed from the Quartet Data Portal (https://chinese-quartet.org/) under the Administrative Regulations of the People’s Republic of China on Human Genetic Resources. They can also be accessed from the Genome Sequence Archive (GSA), Genome Variation Map (GVM), and Open Archive for Miscellaneous Data (OMIX) of the National Genomics Data Center of China with BioProject ID of PRJCA012423 (https://

March 2021

ngdc.cncb.ac.cn/bioproject/browse/PRJCA012423). In addition, source data for data analysis and figure generation are deposited in Zenodo (https://doi.org/10.5281/zenodo.8185817).

Human research participants

Policy information about studies involving human research participants and Sex and Gender in Research.

Reporting on sex and gender	We used blood samples from four participants from a Chinese Quartet family from the Fudan Taizhou Cohort, including father (F7), mother (M8) and monozygotic twin daughters (D5 and D6). Sex and gender of the participants are F7 (male), M8 (female), D5 (female) and D6 (female). Sex and gender information was determined based on self-reporting and DNA sequencing.
Population characteristics	All four participants are adults from Han Chinese. No additional covariates-relevant population characteristics were collected for each donor due to IRB approval restrictions.
Recruitment	The participants were recruited by advertisements in the Taizhou Longitudinal Study. We randomly selected a family with monozygotic twin daughters. No self-selection bias was expected to be introduced.
Ethics oversight	This study was approved by the Institutional Review Board (IRB) of the School of Life Sciences, Fudan University (BE2050). It was conducted under the principles of the Declaration of Helsinki.

Note that full information on the approval of the study protocol must also be provided in the manuscript.

Field-specific reporting

Please select the one below that is the best fit for your research. If you are not sure, read the appropriate sections before making your selection.

☒ Life sciences ☐ Behavioural & social sciences ☐ Ecological, evolutionary & environmental sciences

For a reference copy of the document with all sections, see nature.com/documents/nr-reporting-summary-flat.pdf

Life sciences study design

All studies must disclose on these points even when the disclosure is negative.

Sample size	A total of 108 DNA samples were subjected to four short-read (Illumina HiSeq and NovaSeq, MGI MGISEQ-2000 and DNBSEQ-T7) at six labs for the characterization of small variants. Additionally, 12 DNA samples were measured on three long-read (Oxford Nanopore Technologies (ONT), Pacific Biosciences (PacBio) Sequel and Sequel II) sequencing platforms at three labs to investigate SVs. Epigenomic (methylomic) data involving 72 DNA samples were obtained through Illumina EPIC (850K) array at two labs. RNA sequencing data of 252 samples were generated on MGI DNBSEQ-T7 and Illumina NovaSeq using poly(A) selection or RiboZero library preparation protocols at eight labs. Small RNA sequencing data of 72 samples were generated on Illumina NovaSeq and HiSeq 2500 at four labs. Proteins (annotated from peptides) of 384 samples were measured on nine LC-MS/MS based proteomics platforms (Thermo Scientific Q Exactive, Q Exactive-HF, Q Exactive-HFX, Q Exactive-Plus, Orbitrap Fusion Lumos Tribrid, Orbitrap Fusion, Orbitrap Exploris 480, Bruker timsTOF, and SCIEX Triple TOF6600) at 16 labs. Metabolites of 264 samples were measured on five LC-MS/MS based metabolomics platforms (Thermo Scientific Q Exactive, SCIEX Triple TOF6600, QTRAP 6500+, QTRAP 5500, and Xevo TQ-S) at six labs.
Data exclusions	All data from planed experiments have been included.
Replication	In this study, a batch is defined as 12 libraries from a standard sample set, consisting of 12 vials with each representing one of the triplicates of the Quartet reference sample groups. Except for the long-reads sequencing platforms, the reference materials were profiled within a batch in a lab in 3 replicates for each of the 4 samples (donors). For long-reads sequencing, one replicate for each reference material was sequenced.
Randomization	Aliquots of DNA, RNA, proteins, and metabolites from the same lot were randomly distributed to each center.
Blinding	Each batch of samples distributed was blinded to avoid specific experimental sequences affecting the objective assessment of lab proficiency.

Reporting for specific materials, systems and methods

We require information from authors about some types of materials, experimental systems and methods used in many studies. Here, indicate whether each material, system or method listed is relevant to your study. If you are not sure if a list item applies to your research, read the appropriate section before selecting a response.

Materials & experimental systems

n/a	Involved in the study
☒	☐ Antibodies
☐	☒ Eukaryotic cell lines
☒	☐ Palaeontology and archaeology
☒	☐ Animals and other organisms
☒	☐ Clinical data
☒	☐ Dual use research of concern

Methods

n/a	Involved in the study
☒	☐ ChIP-seq
☒	☐ Flow cytometry
☒	☐ MRI-based neuroimaging

Eukaryotic cell lines

Policy information about cell lines and Sex and Gender in Research

Cell line source(s)	The human immortalized B-lymphoblastoid cell lines of four healthy volunteers from a family Quartet, as part of the Taizhou Longitudinal Study in Taizhou, Jiangsu, China. The sex of the primary cell lines generated from human participants are: F7 (male), M8 (female), D5 (female) and D6 (female).
Authentication	The cell lines have been authenticated by STR profile, karyotype, PCR mycoplasma and sterility testing.
Mycoplasma contamination	No mycoplasma contamination found.
Commonly misidentified lines (See ICLAC register)	No commonly misidentified cell lines were used in this study.
